# Regeneration of the zebrafish retinal pigment epithelium after widespread genetic ablation

**DOI:** 10.1371/journal.pgen.1007939

**Published:** 2019-01-29

**Authors:** Nicholas J. Hanovice, Lyndsay L. Leach, Kayleigh Slater, Ana E. Gabriel, Dwight Romanovicz, Enhua Shao, Ross Collery, Edward A. Burton, Kira L. Lathrop, Brian A. Link, Jeffrey M. Gross

**Affiliations:** 1 Department of Ophthalmology, Louis J Fox Center for Vision Restoration, University of Pittsburgh School of Medicine, Pittsburgh, Pennsylvania, United States of America; 2 Center for Biomedical Research Support, The University of Texas at Austin, Austin, Texas, United States of America; 3 Pittsburgh Institute for Neurodegenerative Diseases, University of Pittsburgh, Pittsburgh, Pennsylvania, United States of America; 4 Department of Neurology, University of Pittsburgh School of Medicine, Pittsburgh, Pennsylvania, United States of America; 5 Tsinghua University Medical School, Beijing, China; 6 Department of Neurobiology and Anatomy, Medical College of Wisconsin, Milwaukee, Wisconsin, United States of America; 7 Geriatric Research, Education and Clinical Center, Pittsburgh VA Healthcare System, Pittsburgh, Pennsylvania, United States of America; 8 Department of Bioengineering, University of Pittsburgh Swanson School of Engineering, Pittsburgh, Pennsylvania, United States of America; 9 Department of Developmental Biology, University of Pittsburgh School of Medicine, Pittsburgh, Pennsylvania, United States of America; Stanford University School of Medicine, UNITED STATES

## Abstract

The retinal pigment epithelium (RPE) is a specialized monolayer of pigmented cells within the eye that is critical for maintaining visual system function. Diseases affecting the RPE have dire consequences for vision, and the most prevalent of these is atrophic (dry) age-related macular degeneration (AMD), which is thought to result from RPE dysfunction and degeneration. An intriguing possibility for treating RPE degenerative diseases like atrophic AMD is the stimulation of endogenous RPE regeneration; however, very little is known about the mechanisms driving successful RPE regeneration *in vivo*. Here, we developed a zebrafish transgenic model (*rpe65a*:nfsB-eGFP) that enabled ablation of large swathes of mature RPE. RPE ablation resulted in rapid RPE degeneration, as well as degeneration of Bruch’s membrane and underlying photoreceptors. Using this model, we demonstrate for the first time that zebrafish are capable of regenerating a functional RPE monolayer after RPE ablation. Regenerated RPE cells first appear at the periphery of the RPE, and regeneration proceeds in a peripheral-to-central fashion. RPE ablation elicits a robust proliferative response in the remaining RPE. Subsequently, proliferative cells move into the injury site and differentiate into RPE. BrdU incorporation assays demonstrate that the regenerated RPE is likely derived from remaining peripheral RPE cells. Pharmacological disruption using IWR-1, a Wnt signaling antagonist, significantly reduces cell proliferation in the RPE and impairs overall RPE recovery. These data demonstrate that the zebrafish RPE possesses a robust capacity for regeneration and highlight a potential mechanism through which endogenous RPE regenerate *in vivo*.

## Author summary

Diseases resulting in retinal pigment epithelium (RPE) degeneration are among the leading causes of blindness worldwide, and no therapy exists that can replace RPE or restore lost vision. One intriguing possibility is the development of therapies focused on stimulating endogenous RPE regeneration. For this to be possible, we must first gain a deeper understanding of the mechanisms underlying RPE regeneration. Here, we develop a transgenic zebrafish system through which we ablate large swathes of mature RPE and demonstrate that zebrafish regenerate RPE after widespread injury. Injury-adjacent RPE proliferate and regenerate RPE, suggesting that they are the source of regenerated tissue. Finally, we demonstrate that Wnt signaling may be involved in RPE regeneration. These findings establish a versatile *in vivo* model through which the molecular and cellular underpinnings of RPE regeneration can be further characterized.

## Introduction

The RPE is a polarized monolayer of pigment-containing cells that separates the retina from the choroid and performs many critical functions for vision. Microvilli extend from the apical RPE surface and interdigitate with photoreceptor outer segments, enabling the RPE to support photoreceptor health [1]. The basal surface of the RPE abuts and helps to form Bruch’s membrane (BM), which, along with tight junctions between RPE cells, creates the blood-retina barrier and facilitates nutrient and ion transport between the retina and choriocapillaris [2–4]. Additionally, RPE pigment prevents light scatter by absorbing stray photons. Due to its importance in maintaining retinal function, diseases affecting the RPE have dire consequences for vision. Age-related macular degeneration (AMD) is one such disease, and is the third leading cause of blindness in the world [5,6]. AMD is commonly divided into two types: atrophic (dry) and exudative (wet). In the early stages of atrophic AMD, RPE cells in the parafovea become dysfunctional and progressively degenerate, and this is thought to result in death of parafoveal rods [7–9]. Progressively, RPE dysfunction and degeneration spread to the fovea, resulting in loss of cone photoreceptors, and ultimately, loss of high-acuity vision [10–12]. Exudative AMD occurs in a subset of atrophic AMD cases when choroidal vasculature invades the retina [11,13].

Transplantation of stem cell-derived RPE has emerged as a possibility for treating AMD [14–16], and clinical trials are currently underway [17–23]. However, little is known about the fate of transplanted RPE, and whether their survival and integration can be improved. An unexplored complementary approach is the development of therapies that stimulate endogenous RPE regeneration. In mammals, RPE regeneration is limited and dependent upon the size of the injury [24]; small lesions can be repaired by the expansion of adjacent RPE [25,26], but existing RPE are unable to repair large lesions [24,27–30]. In some injury paradigms, RPE cells proliferate but do not regenerate a morphologically normal monolayer (e.g. [26,31,32]). Indeed, RPE often overproliferate after injury, such as during proliferative vitreoretinopathy (PVR), where proliferative RPE invade the subretinal space and lead to blindness [33–35]. Recently, a subpopulation of quiescent human RPE stem cells was identified that can be induced to proliferate *in vitro* and differentiate into RPE or mesenchymal cell types [30,36], suggesting that the human RPE contains a population of cells that could be induced to regenerate.

Little is known about the process by which RPE cells respond to elicit a regenerative, rather than pathological, response. Indeed, no studies have demonstrated regeneration of a functional RPE monolayer following severe damage in any model system. The development of such a model is a critical first step to acquiring a deeper understanding of the molecular mechanisms underlying RPE regeneration. Zebrafish offer distinct advantages for this purpose: the development, structure and function of the zebrafish eye is similar to human, including a cone-rich larval retina; they are amenable to genetic manipulation and imaging, and they can regenerate neural tissues (e.g.[37–39]). However, it is unknown whether the zebrafish RPE is capable of regeneration. Here, we demonstrate that the zebrafish RPE possesses a robust capacity for regeneration and identify cellular and molecular mechanisms through which endogenous RPE regenerate *in vivo*.

## Results

### RPE ablation results in photoreceptor degeneration

**Fig 1:**
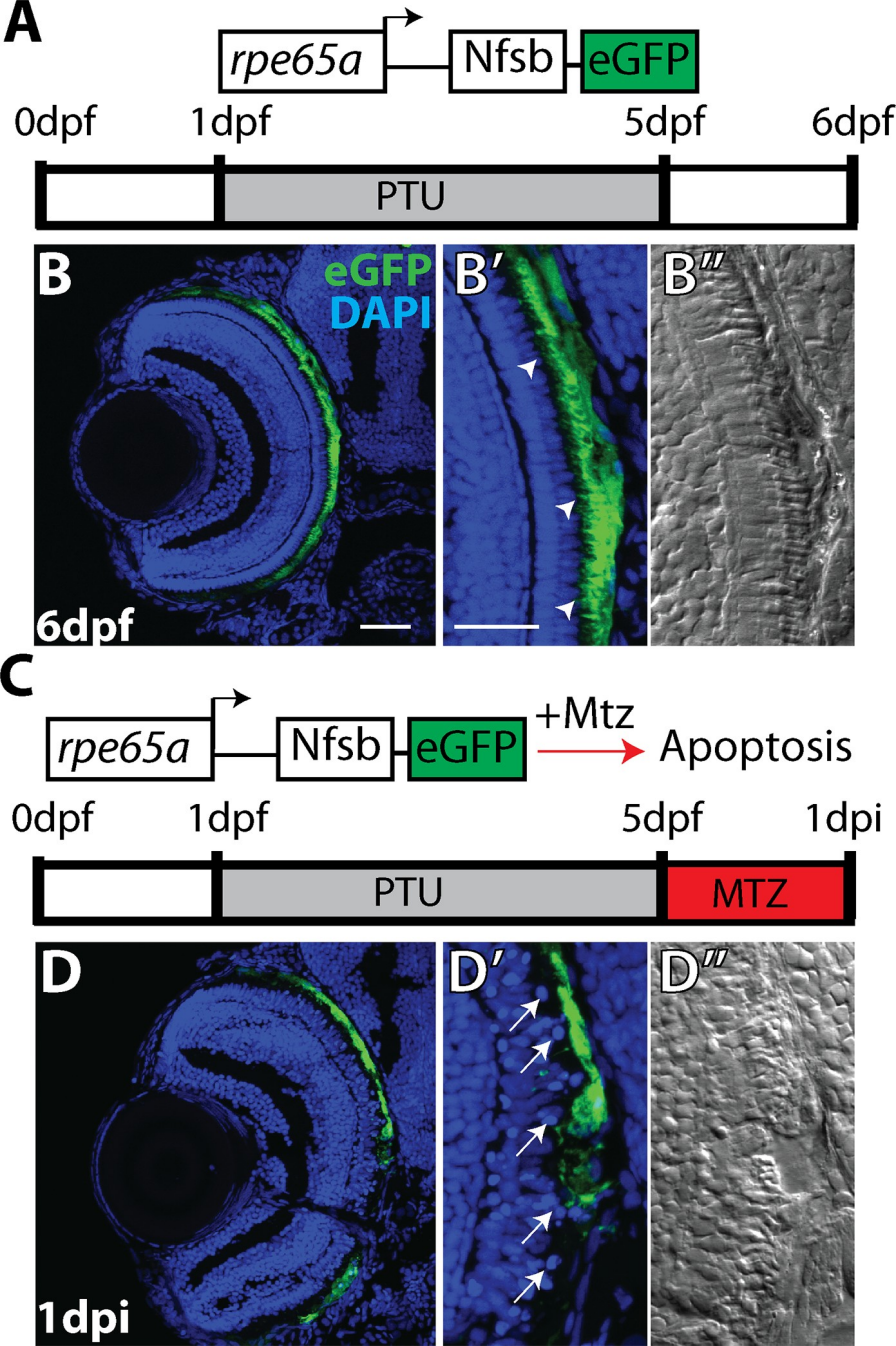
RPE ablation paradigm. (A) Cartoon depicting the *rpe65a*:nfsB-eGFP transgene and treatment course of unablated embryos. (B) Transverse cryosections of an unablated 6dpf larva. (B,B’) After exposure to PTU between 1-5dpf, transgene expression is specifically restricted to mature RPE cells, with the brightest expression confined to the central two-thirds of the RPE. Arrowheads indicate apical microvilli. (B”) DIC images reveal RPE repigmentation and normal photoreceptor layer architecture. (C) Cartoon depicting the nitroreductase-mediated ablation paradigm: after washing out PTU, larvae were treated with MTZ for 24 hours. Within cells expressing the transgene, nfsB converts MTZ into a potent DNA crosslinking agent and induces cell death. (D,D’)Transverse cryosections of a 1dpi larva reveal significant disruption of eGFP^+^ cell morphology and disorganization in INL nuclear lamination. Arrows indicate delaminated and pyknotic nuclei. (D”) DIC images reveal a lack of RPE pigmentation and the marked disruption of photoreceptor layer architecture. Green = eGFP, blue = nuclei. Dorsal is up and distal is left. Scale bar = 40μm.

To develop an RPE injury model, we utilized a transgenic line in which a promoter element from *rpe65a* drives expression of the nfsB-eGFP fusion protein in mature RPE [40] (*rpe65a*:nfsB-eGFP; Fig 1). nfsB is an *E*. *coli* nitroreductase that converts the ordinarily benign prodrug metronidazole (MTZ) into a potent DNA crosslinking agent, leading to apoptosis in expressing cells [41–44]. *rpe65a*:nfsB-eGFP transgenic embryos were treated with phenylthiourea (PTU) [45] to suppress melanin synthesis. To ablate RPE, 5dpf larvae were removed from PTU and exposed to 10mM MTZ for 24 hours. After treatment, eGFP^+^ cells degenerate (Fig 1D), nuclei in the outer nuclear layer (ONL) adjacent to ablated RPE become disorganized (Fig 1D’) and photoreceptor outer segment morphology is disrupted (Fig 1D”). Degeneration of eGFP^+^ cells was accompanied by the absence of pigmentation recovery after removal of PTU. To quantify this, eyes were enucleated from ablated and control larvae, and brightfield images were taken to provide an *en face* view of the RPE (S1 Fig). Quantification of the mean pigment intensity showed that pigmentation in ablated eyes was significantly reduced compared to controls by 2dpi (p<0.0001).

**Fig 2:**
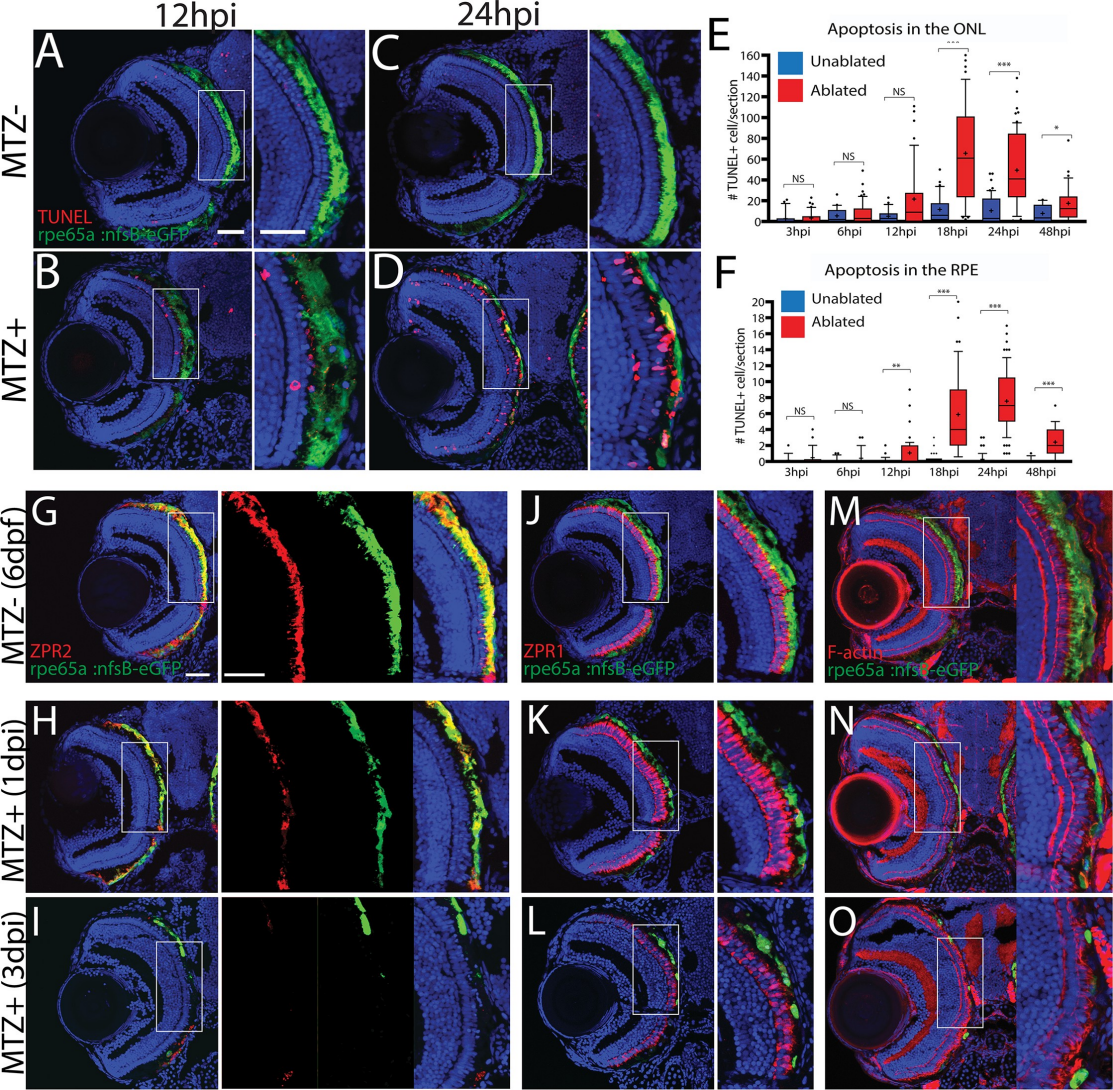
Ablation of the RPE leads to degeneration of underlying photoreceptors. (A-D) Transverse cryosections stained for TUNEL (red). Compared to untreated (A,C) larvae, ablated RPE were disrupted by 12hpi (B), and TUNEL^+^ cells appeared throughout the RPE and ONL at 24hpi (D). (E, F) Quantification of TUNEL^+^ cells/section in the RPE (E) and ONL (F) revealed a significant increase in the RPE by 12hpi and in the ONL by 18hpi. Significance determined using Mann-Whitney U test. * p≤0.05, ** p<0.005, *** p<0.0005. (G-I) Transverse sections of unablated 6dpf larvae stained for ZPR2 (G), ZPR1 (J), and F-Actin (M) (red). By 1dpi, ZPR2 is disrupted in a similar manner to eGFP (H), and ZPR1^+^ cones appear swollen and degenerated (K), and photoreceptor outer segment cytoskeletons become disorganized (N). By 3dpi, ZPR2 signal is absent from the central injury site (I) and PR morphology is notably degraded (L,O). Green = eGFP, blue = nuclei. Dorsal is up and distal is left. Scale bar = 40μm.

To characterize the temporal dynamics of RPE and photoreceptor (PR) degeneration following MTZ treatment, sections were taken from larvae at 3, 6, 12, 18, 24 and 48 hours post-injury (hpi) and stained for TUNEL (Fig 2; S2 Fig). At 3hpi, TUNEL^+^ nuclei were detected in the RPE (S2A and S2B Fig) while the ONL appeared normal. At 6hpi, nuclear organization in the ONL began to deteriorate and by 12hpi, apoptosis significantly increased in the RPE (Fig 2E, p = 0.016) and ONL nuclei became delaminated. By 18hpi, apoptosis in the ONL increased significantly (Fig 2F, p<0.0001) and eGFP accumulated in blebs, a process which left regions of the RPE devoid of eGFP signal (S2G and S2H Fig). RPE apoptosis peaked at 24hpi (Fig 2E, p<0.0001). Apoptosis, while remaining significantly elevated when compared to controls, began to decrease in both layers by 48hpi (Fig 2E and 2F, p<0.0001 in RPE, p = 0.0301 in ONL). However, by 48hpi, all remaining eGFP signal was contained in irregular eGFP^+^ blebs, likely consisting of RPE cell debris. ONL nuclear lamination remained severely disrupted (S2I–S2L Fig). Non-transgenic siblings treated with MTZ showed no significant increase in apoptosis (S3 Fig).

To characterize degeneration further, RPE-ablated larvae were stained with markers for RPE (ZPR2) [46], red/green cone arrestin (ZPR1) [47], and F-actin (phalloidin) (Fig 2G–2O). In unablated larvae, nfsB-eGFP colocalized with ZPR2 in the central RPE, confirming fidelity of the transgene (Fig 2G). *rpe65a*:nfsB-eGFP was expressed in mature RPE cells while ZPR2 signal extended further into the periphery, labeling both mature eGFP^+^ RPE as well as less-mature eGFP^-^ RPE closer to the ciliary marginal zone (CMZ) (Fig 2G). Between 1 and 3 days post-injury (dpi), changes to ZPR2 staining recapitulated disruption of eGFP^+^ RPE, including degeneration of the RPE cell body. ZPR1^+^ cones also began degenerating at 1dpi (Fig 2J), and F-actin bundles in photoreceptor outer segments became more diffuse and lost their perpendicular orientation (Fig 2M). By 3dpi, both eGFP and ZPR2 signals were absent from the central RPE, confirming degeneration of RPE in the central injury site (Fig 2I). PR degeneration in the central retina also peaked at this time, displaying aberrant cone morphology (Fig 2L), and significant degeneration of photoreceptor outer segments throughout the injury site (Fig 2O). Despite rigorous screening, some variability in ablation severity was observed, likely from variations in transgene expression and ablation efficiency. To mitigate variability, only larvae with high levels of eGFP signal disruption in the eye (severe ablation) were utilized in subsequent experiments. In severely ablated larvae, ablation-mediated degeneration reliably peaked between 1-2dpi (i.e. as in Fig 2).

**Fig 3:**
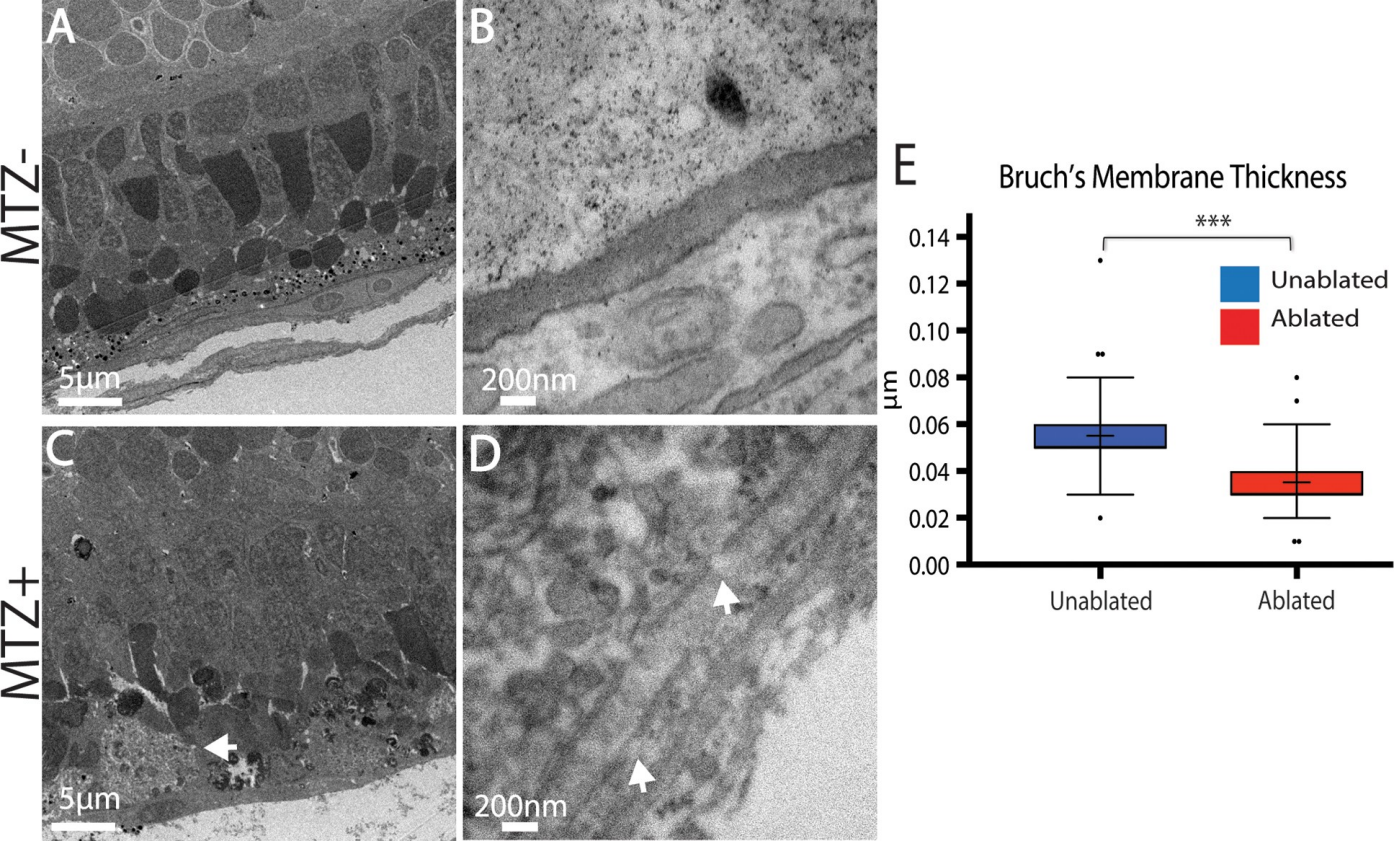
TEM analysis confirms degeneration of the RPE, photoreceptors, and Bruch’s Membrane. (A,B) TEM images of unablated 8dpf and (C,D) 3dpi eyes. Compared to unablated controls, the ONL and RPE is degenerated in ablated larvae, with large aggregates of debris notable in the RPE (C, arrow). Magnified views of BM reveal reduced BM thickness as well as obvious gaps (D, arrows). (E) Quantification of BM thickness reveals a significant reduction in BM thickness in ablated larvae (Student’s T-test, MTZ- n = 3 eyes, MTZ+ n = 4 eyes p<0.0001).

Immunohistochemical data strongly supported RPE and PR degeneration following ablation and this was confirmed by transmission electron microscopy (TEM) analyses (Fig 3). In unablated larvae, central RPE cells containing pigmented melanosomes were easily observable (Fig 3A). The PR layer was also properly laminated and contained readily identifiable cone and rod outer segments (Fig 3A). Analysis of ablated larvae at 3dpi revealed severe degeneration of the RPE, which was occupied by debris that was either distributed throughout the injury site or collected in membrane-enclosed structures that may be macrophages (Fig 3C, arrow). Bruch’s membrane (BM) underlying the ablated RPE was also significantly thinner than in controls (Fig 3B, 3D and 3E; p<0.0001) and contained obvious gaps (Fig 3D). Consistent with defects detected by histology (Fig 2), the PR layer of ablated larvae was severely degenerated, showing reduced size and integrity of photoreceptor outer segments, and containing degenerated outer segment material and other cellular debris (Fig 3C).

Taken together, these data indicate that RPE ablation via the *rpe65a*:nfsB-eGFP transgene causes specific loss of central RPE cells in larval zebrafish, with morphological defects beginning at 3hpi and destruction of the central RPE peaking at 2dpi. Further, immunohistological analyses demonstrate that underlying photoreceptor cells also degenerate rapidly after RPE ablation.

### Ablation of the RPE leads to defects in functional vision

**Fig 4:**
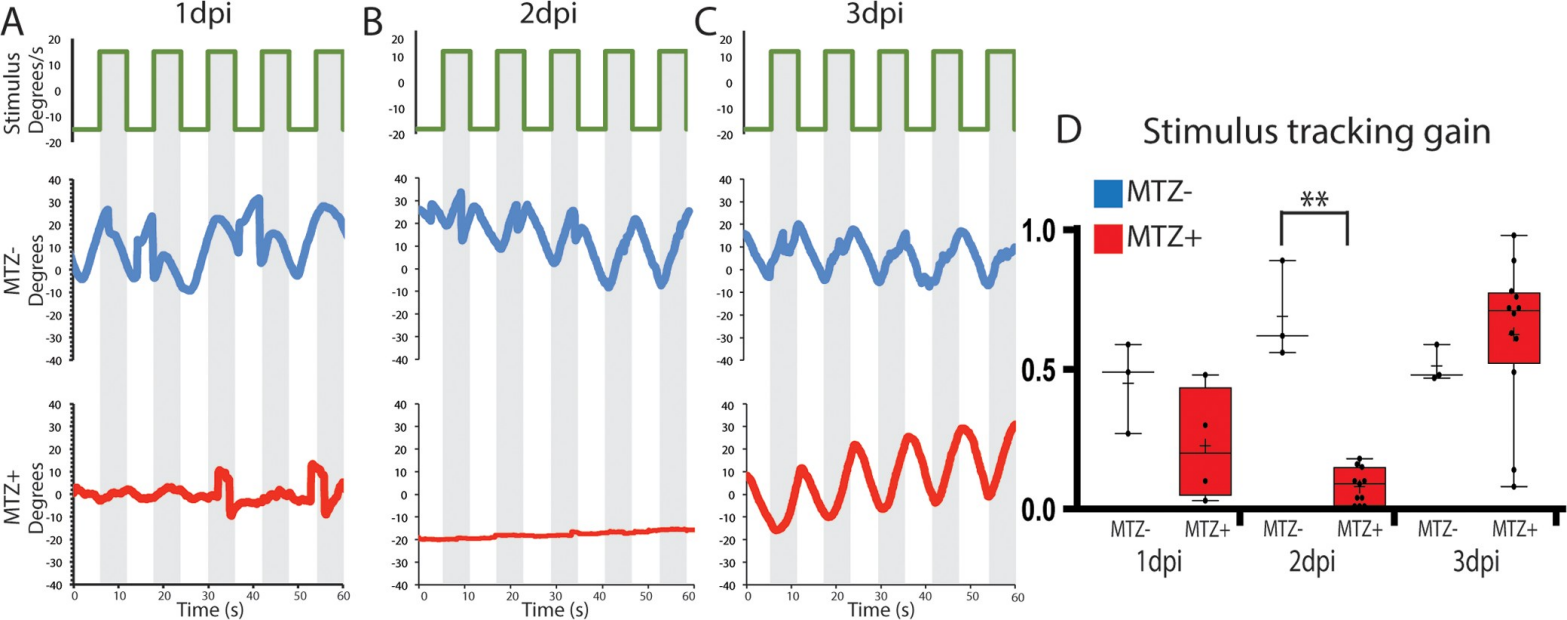
RPE ablation results in defects in visual function. (A-C) To measure the OKR, the right eye of ablated and unablated larvae was exposed to a rotating stimulus, and the position of the stimulated eye was recorded at 1dpi (A, n = 3 unablated, n = 4 ablated), 2dpi (B, n = 3 unablated, n = 12 ablated) and 3dpi (C, n = 3 unablated, n = 12 ablated). (D) Quantification revealed that ablated larvae had a significantly reduced stimulus tracking gain at 2dpi (Mann-Whitney U test, P = 0.0055). Recovered OKR was detectible by 3dpi (Mann Whitney U test, P = 0.1878).

Visual function of larvae was evaluated by analyzing the optokinetic response (OKR) to determine whether ablation of the RPE results in vision defects [48–50] (Fig 4). A cohort of ablated and control larvae were exposed to a rotating full-field visual stimulus at 1dpi, 2dpi and 3dpi, and visual responses were recorded (Fig 4A–4C). At 1dpi, ablated larvae exhibited a modest reduction in stimulus tracking gain relative to controls, and this reduction in gain became significant at 2dpi (Fig 4D, p = 0.0055) indicating that visual function is disrupted after ablation. By 3dpi, ablated larvae demonstrated a recovery of stimulus tracking gain (Fig 4C and 4D). Likely, this rapid recovery is due to new photoreceptors being generated from the continually proliferative CMZ (*see below*). Collectively, these data demonstrate that ablation of large swathes of mature RPE cells in *rpe65a*:nfsB-eGFP transgenics results in the rapid degeneration of underlying PRs and BM, and a loss of visual function.

### Regeneration of the RPE occurs in a peripheral-to-central fashion

**Fig 5:**
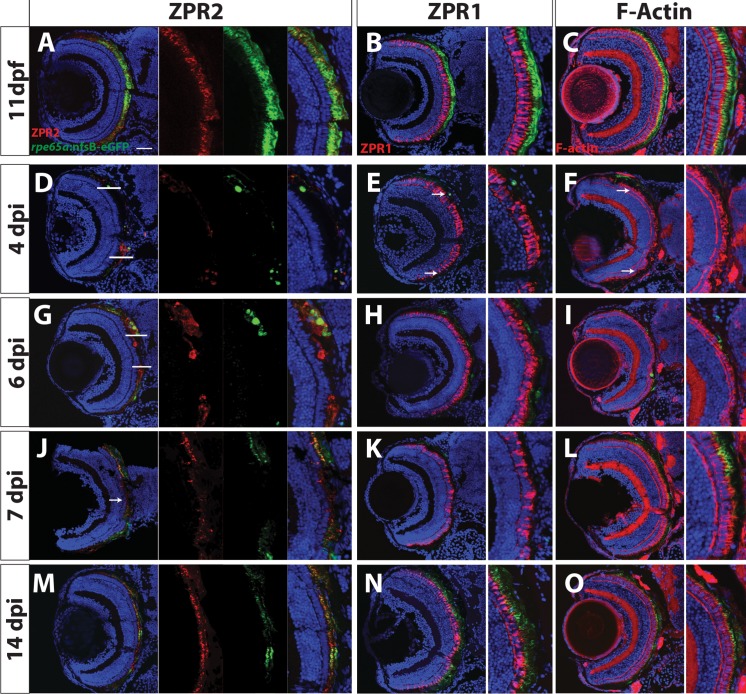
RPE regeneration initiates in the periphery and proceeds inward. Transverse sections of unablated larvae stained for the RPE marker ZPR2 (A), R/G cone photoreceptor marker ZPR1 (B) and F-Actin (C) at 11dpf. Ablated eyes stained for ZPR2 (D,G,J,M), ZPR1 (E,H,K,N), and Phalloidin (F,I,L,O) at 4, 6, 7 and 14dpi. Green = eGFP, blue = nuclei, red = marker. eGFP^+^ RPE appears in the periphery at 4dpi (marked by arrows in D-F). As regeneration proceeds, eGFP^+^ RPE extends further toward the eye center, and the leading tip of the regenerated monolayer often consists of both immature and mature RPE (ZPR2^+^/eGFP^-^ cells in G). PR morphology appears to recover in the periphery proximal to regenerated RPE. By 7dpi, ZPR2^+^ RPE is present throughout the RPE (J), and PR morphology begins to recover in the central injury site (K,L). By 14dpi, mature eGFP^+^/ZPR2^+^ RPE cells are present throughout the RPE (M), and PR morphology further improves in the central retina (N,O). Dorsal is up and distal is left. Scale bar = 40μm.

**Fig 6:**
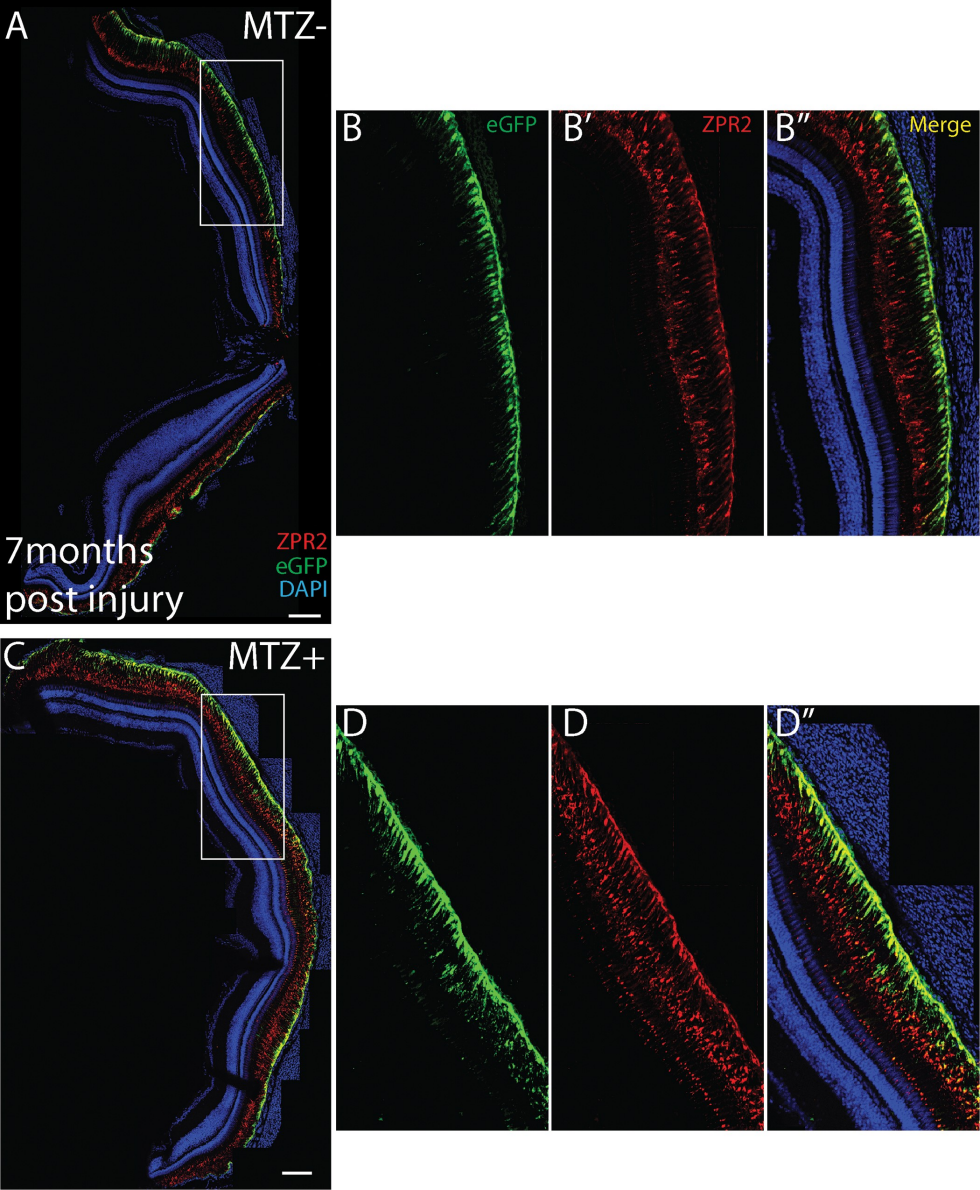
Regenerated RPE appears normal 7 months post-injury. Stitched-together confocal images of transverse cryosections of 7 month post-injury fish (C,D) and age-matched sibling controls (A-B). (A) Robust eGFP and ZPR2 expression exists throughout the central RPE. (B-B”) Within the central RPE, eGFP is strongly expressed in the RPE cell body and labels apical processes, most strongly toward the cell body, while ZPR2 labels the RPE cell body and apical processes closer to the outer limiting membrane. (C) In ablated fish, strong expression of eGFP and ZPR2 is evident throughout the retina. (D-D”), and a similar pattern of eGFP and ZPR2 expression is observed in the central injury site. Scale bar = 100μm.

As discussed above, a subset of RPE possess a latent ability to proliferate *in vitro* [36] and various degrees of RPE repair have been documented (e.g. [25,26,31,32,51,52]) but in none of these systems is the RPE able to recover a functional monolayer following a large injury. Zebrafish possess a remarkable ability to regenerate a multitude of tissues [37,38,53], but it is unknown if they can regenerate RPE. Thus, we analyzed the regenerative capacity of ablated larvae at 4, 6, 7, and 14dpi with ZPR2, ZPR1, and phalloidin (Fig 5). At 4dpi, ZPR2^+^ cells extended into the injury site (Fig 5D) and RPE pigmentation significantly increased when compared to 2dpi levels (S1 Fig), suggesting that RPE cells have begun to regenerate. Although ZPR1-labeled cones and photoreceptor outer segments remained degenerated in the central ablation site, morphologically normal ZPR1-positive cones reappeared in the periphery, and these were always in direct apposition to regenerated eGFP^+^ RPE (Fig 5E and 5F; S4 Fig). At 6dpi, morphologically normal eGFP^+^/ZPR2^+^ RPE cells populate the periphery and approach the central injury site (Fig 5G), and PR morphology improves in a similar pattern (Fig 5H and 5I; S4 Fig). Interestingly, ZPR2^+^/eGFP^-^ cells always appeared at the advancing tip of the regenerating monolayer (Fig 5G). While the *rpe65a*:nfsB-eGFP transgene is expressed specifically in mature RPE, ZPR2 labels less-mature RPE, suggesting that these ZPR2^+^/eGFP^-^ cells are RPE that have not yet fully differentiated. By 7dpi, the injury site was populated by ZPR2^+^ RPE (Fig 5J). Although ZPR1-labeled cones continued to possess aberrant outer segment morphologies compared to controls in the central retina at 7dpi (Fig 5K), photoreceptor outer segment architecture began to improve at this time (Fig 5L). By 14dpi, ZPR2^+^/eGFP^+^ cells populated the entire RPE layer, and these displayed proper RPE cell morphology (Fig 5M and 5N). While most ZPR1^+^ cones displayed proper morphology, ONL disorganization persisted, particularly in the injury site, where cones failed to align perpendicularly to the RPE (Fig 5N). Seven months post-ablation, the RPE of ablated larvae were morphologically similar to those in unablated siblings (Fig 6).

**Fig 7:**
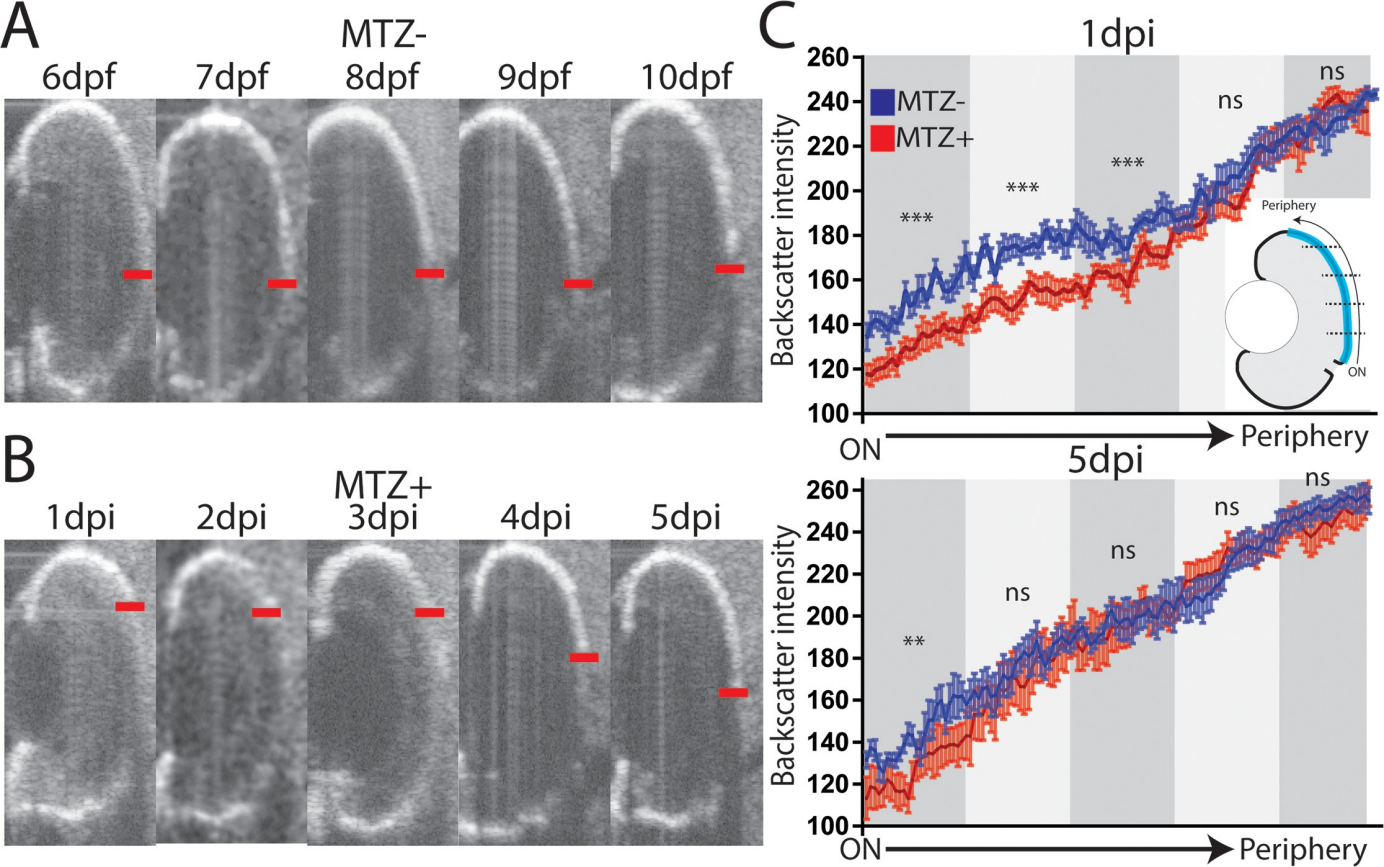
Longitudinal OCT analysis of RPE regeneration. (A-C) Representative time series from a single (A) unablated and (B) ablated larva. Central edge of maximal RPE intensity marked with red line. (C) Quantification of the RPE signal (backscatter) from the dorsal periphery to optic nerve across unablated (blue) and ablated (red) larvae at 1dpi and 5dpi (error bars = SEM). The measured RPE was divided into quintiles (cartoon), and the area under the curve within each quintile was measured. At 1dpi, backscatter intensity in ablated RPE is significantly below unablated intensity in the 3 quintiles closest to the optic nerve, while at 5dpi, only the central-most quintile is significantly reduced (MTZ^-^: n = 10, MTZ^+^ n = 9, Student’s unpaired t test, **p<0.005, ***p<0.0005).

Regeneration appeared to proceed in a periphery-to-center fashion in fixed samples. We utilized optical coherence tomography (OCT) to quantify the spatial and temporal dynamics of RPE degeneration and regeneration in individual larva over time. The RPE in OCT images presents as a bright line due to the density and pigment present in intact tissue; in ablated eyes, the intensity of the signal decreases as a result of tissue disruption (Fig 7A and 7B). Intensity of RPE signal (backscatter) can be quantified by determining the pixel intensity at each position of the RPE; here, we quantified the intensity from the optic nerve to the dorsal periphery, and examined changes in intensity in individual larvae over time (Fig 7, S1–S6 Videos). This analysis revealed that backscatter was significantly decreased in ablated larvae compared to controls in the central-most three quintiles of the RPE at 1dpi, and that all but the central-most quintile recovered to unablated levels by 5dpi (Fig 7C, p<0.0001). These results further support a model in which RPE regeneration occurs in a peripheral-to-central manner.

**Fig 8:**
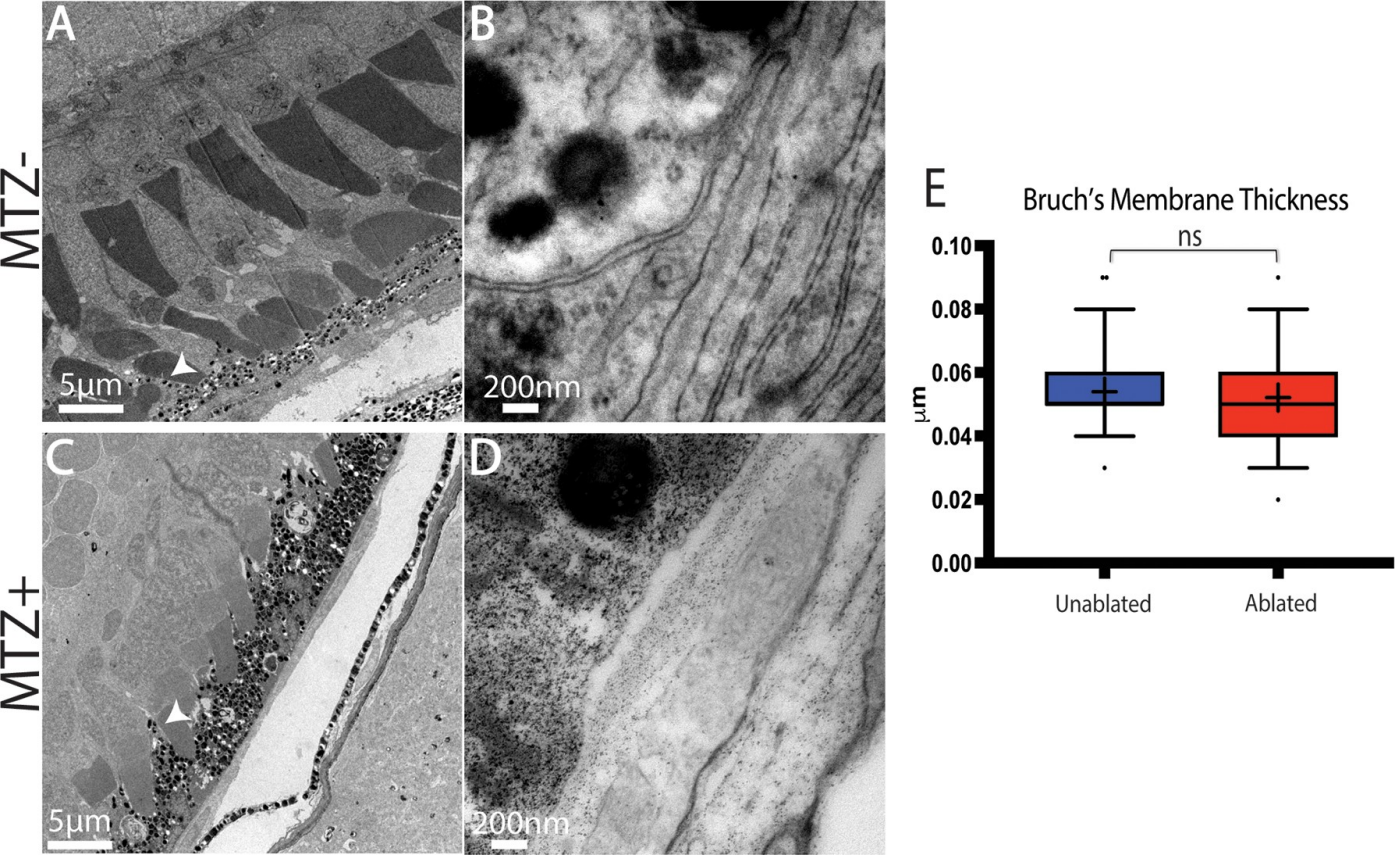
TEM analysis of regenerated RPE. (A,B) TEM images of unablated 19dpf and (C,D) 14dpi eyes (C) Organized photoreceptor outer segments are visible in the ablated photoreceptor layer, and a regenerated RPE is present. (E) Quantification of BM thickness. Student’s T-test reveals that BM thickness is not significantly different in ablated larvae * p<0.05. (MTZ^-^ n = 3 eyes, 81 measurements; MTZ^+^ n = 3, 81 measurements).

To confirm that the regenerated RPE is morphologically normal, TEM analyses were performed on 14dpi larvae (Fig 8), a time point at which eGFP^+^/ZPR2^+^ RPE cells populate the injury site (Fig 5N). Both unablated and ablated RPE contained melanosomes (Fig 8A and 8C). Moreover, BM thickness was restored in ablated larvae (Fig 8B, 8D and 8E; p = 0.3402). Despite this apparent recovery, subtle differences still existed between regenerated and unablated RPE: RPE in the regenerated region appeared to contain more melanosomes and had thicker cell bodies (Fig 8A and 8C). Consistent with immunohistochemical results, at 14dpi ONL lamination was improved but not completely recovered. Taken together, these data demonstrate that larval zebrafish are capable of regenerating a functional RPE monolayer following widespread RPE ablation and that regeneration is rapid, occurring within 1–2 weeks post-ablation.

### Regeneration of the adult RPE

**Fig 9:**
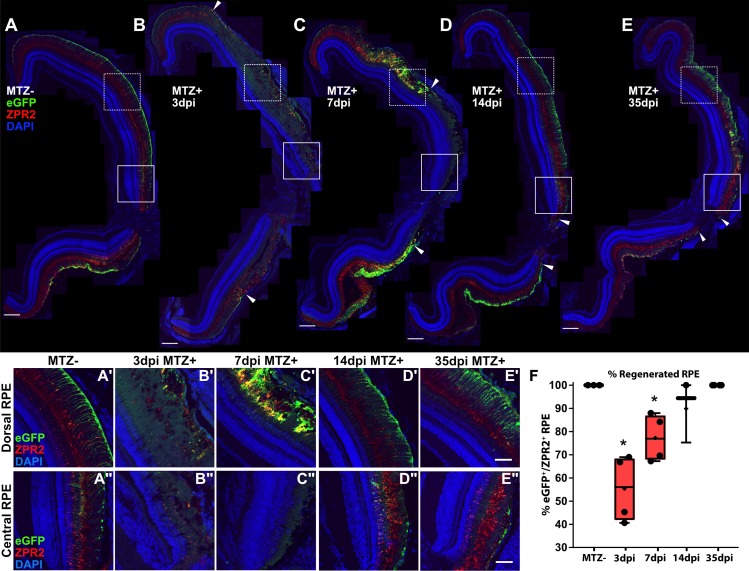
Regeneration of the adult RPE initiates in the periphery and proceeds centrally. (A-E) Stitched-together transverse cryosections of unablated (A, n = 4), 3dpi (B, n = 4), 7dpi (C, n = 4), 14dpi (D, n = 3), and 35dpi (E, n = 4) adult eyes, as well as magnified insets of the dorsal peripheral RPE (A’-E’) and central RPE (A”-E”). Red = ZPR2, Green = eGFP, blue = nuclei. At 3dpi, RPE cell degeneration occurred in a large portion of the RPE (B, *arrowheads*), indicated by loss of eGFP and ZPR2 expression and disruption of overall morphology of the RPE (B’,B”). At 7dpi, increased colocalization of eGFP and ZPR2 defined the peripheral edge of the RPE injury site (C’,C, *arrowheads*) while the central RPE was absent eGFP and ZPR2 expression (C”). At 14dpi, ZPR2^+^/eGFP^+^ RPE reappears in the periphery (D’) and extends inward toward the injury center (D, *arrowheads*). Magnified images of the central injury site also reveal recovered ZPR2 localization to the apical processes (D”). At 35dpi, ZPR2^+^/eGFP^+^ RPE reappear throughout the injury site (E), and proper polarization of ZPR2 and eGFP colocalization has been reestablished throughout (E’-E”). (A-E) Scale bar = 100μm. (A’-E”) Scale bar = 40μm. (F) Quantification of percent RPE regeneration based on measurements of contiguous eGFP^+^/ZPR2^+^ expression revealed significant RPE degeneration at 3dpi and 7dpi when compared to MTZ- controls. RPE recovery occurred by 35dpi. Mann-Whitney U Test, * p<0.05.

In larvae, the eye undergoes significant growth, making it possible that RPE regeneration is the result of a permissive growth environment rather than an ability of the RPE to regenerate *per se*. Thus, we determined whether RPE regeneration also occurs in the adult eye. Transgene expression in unablated adults is restricted to mature central RPE as it is in larvae (Fig 9A). At 3dpi, there were clear signs of RPE degeneration that mirrored those in RPE-ablated larvae, including disruption of cell body cohesion and deterioration of apical processes, as indicated by the aberrant expression of ZPR2 and eGFP (Fig 9B). Degeneration extended from the central RPE (Fig 9B”) to the periphery (Fig 9B’). At 7dpi, adults showed signs of RPE regeneration in the peripheral injury site, such as recovery of contiguous eGFP^+^/ZPR2^+^ RPE (Fig 9C, *arrowheads*; Fig 9C’), however central RPE had not yet recovered (Fig 9C”). By 14dpi (Fig 9D) and 35dpi (Fig 9E), adult zebrafish showed restoration of peripheral eGFP^+^/ZPR2^+^ RPE (Fig 9D’ and 9E’) as well as successful regeneration of central RPE with apically localized ZPR2 expression (Fig 9D” and 9E”), similar to sibling controls (Fig 9A, 9A’ and 9A”). Quantification of RPE recovery based on contiguous eGFP^+^/ZPR2^+^ expression showed significant degeneration occurred by 3dpi (p = 0.0286), and that RPE fully regenerated by 35dpi (Fig 9F). Taken together, these results demonstrate that the adult zebrafish is also capable of regenerating the RPE, and in a similar periphery-to-center mechanism as occurs in larvae (Fig 9B–9E, *arrowheads*). Given these similarities and the technical advantages of using larvae over adults (e.g. comparatively rapid regeneration, access to a large number of samples, the ease of *in vivo* imaging and genetic manipulations, and utility in high-throughput drug screens), we focused further efforts on characterizing the mechanisms underlying RPE regeneration in larvae.

### Dynamics of proliferative cells during RPE regeneration

The rate and periphery-to-center pattern of RPE regeneration suggest that regeneration is driven by cell proliferation, and not simply the expansion of individual RPE cells, a response noted in several systems after small RPE injuries [24,31,54]. Proliferative cells are a major component of regeneration in diverse tissues, and they often derive from a resident pool of progenitor cells, e.g. as in blood and skin [55,56], or from differentiated cells that are stimulated to respond to injury, e.g. as in heart and retina [53,57,58]. Moreover, RPE cell proliferation results from the loss of BM contact in several injury contexts, and pathologically, during PVR [24,30,59,60]. Thus, we hypothesized that uninjured peripheral RPE cells respond to injury by dedifferentiating and proliferating to replace lost tissue.

**Fig 10:**
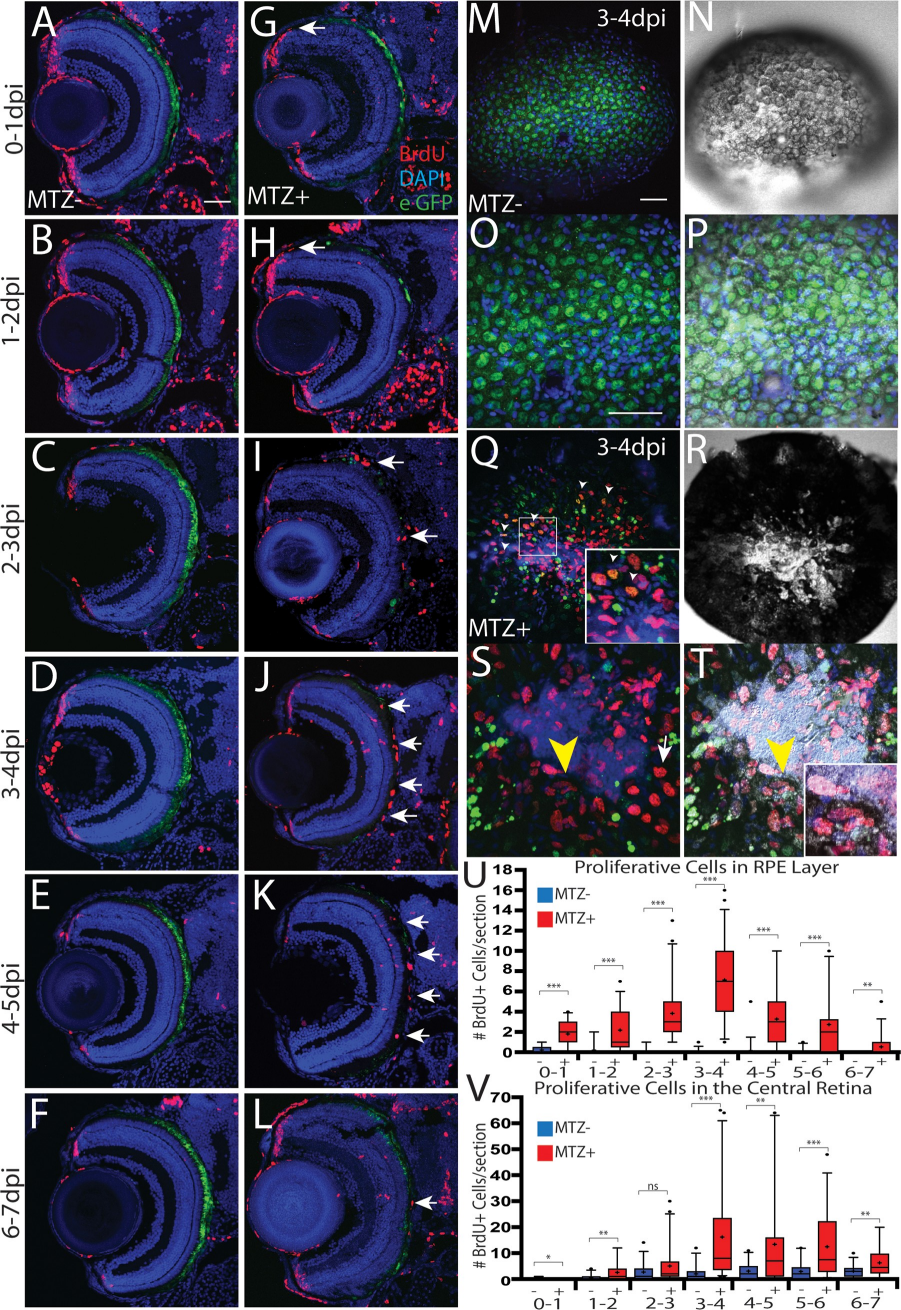
RPE regeneration involves a robust proliferative response. (A-L) Transverse retinal sections of unablated (A-F) and ablated (G-L) larvae exposed to 24-hour BrdU pulses at various days post-injury. BrdU^+^ cells first appear in the periphery as early as 0-1dpi (arrow, G), and 1-2dpi (arrow, H). As regeneration proceeds, BrdU^+^ cells appear closer to the central injury site and at the inner tip of the regenerating RPE layer (arrows, I). BrdU^+^ cells then populate the injury site (arrows, J-L). (M-T) *en face* wholemount images of unablated (M-P) and ablated (Q-T) eyes from larvae exposed to BrdU between 3-4dpi. White arrowheads in (Q) and (Q, inset) indicate BrdU^+^/eGFP^+^ cells near the injury site. Yellow arrowhead in (S) and (T) indicate BrdU^+^ cells proximal to the injury site that are beginning to become pigmented. (Inset, T) Magnified image of BrdU^+^, pigmented cells. (U) Quantification of total number of BrdU^+^ cells/section in the RPE (U) and central retina (V) reveals an increase of BrdU^+^ cells in the RPE starting at 0-1dpi and peaking at 3-4dpi. Proliferation in the central retina significantly increased at 3-4dpi. Mann-Whitney U Test, * p<0.05, ** p<0.005, *** p<0.0005. Dorsal is up and distal is left. Scale bar = 40μm.

To test this hypothesis, we first performed 24-hour BrdU incorporation assays to characterize the total number of proliferative cells within the RPE layer throughout regeneration (Fig 10). Proliferative cells appeared in the RPE as early as 1dpi, largely appearing immediately adjacent to the CMZ or in the center of the ablation site (Fig 10G and 10U, p<0.0001). Between 2-3dpi, more proliferating cells localized to the center of the eye, within the injury site (Fig 10I), and the number of proliferative cells in the RPE peaked between 3-4dpi (Fig 10J and 10U). During this period, proliferative cells populated much of the central eye in ablated larvae, with many localizing adjacent to or within the injury site (Fig 10J and 10Q–10T). In contrast, unablated eyes showed eGFP^+^ RPE throughout the central RPE (Fig 10D and 10M–10P) and sparse BrdU^+^ nuclei (Fig 10M–10P). As regeneration continued, eGFP^+^ RPE cells appeared closer to the center of the injury site and the number of proliferative cells in the RPE layer decreased (Fig 10L and 10U), with most remaining proliferative cells localizing to the injury site. As expected, BrdU^+^ cells were also observed in the retina, and these are likely Müller glia-derived progenitor cells (MGPCs) generated in response to PR degeneration [58,61,62]. Quantification of the number of BrdU^+^ cells in the central retina demonstrated that the kinetics of retinal regeneration largely overlapped that of the RPE (Fig 10V).

**Fig 11:**
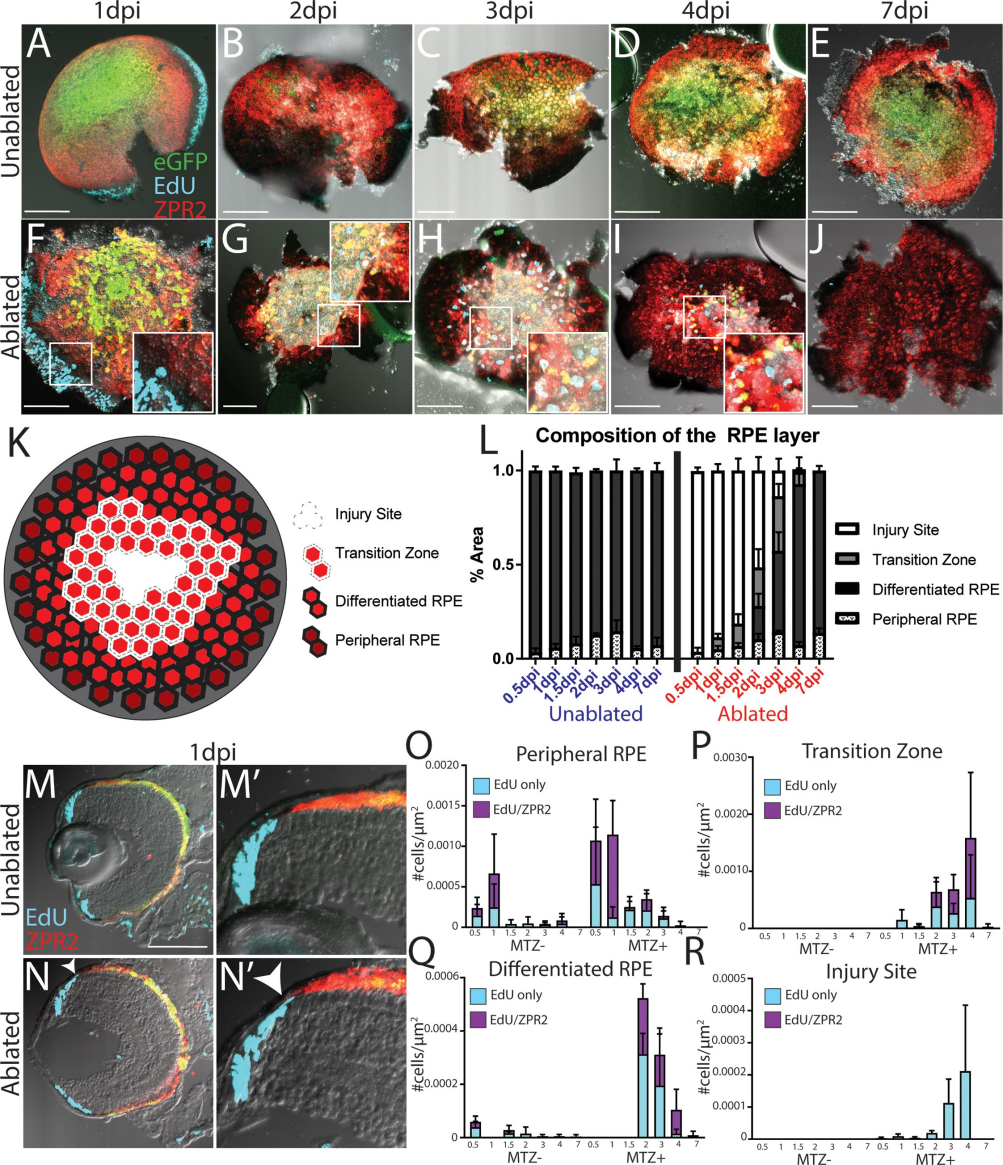
Wholemount analysis of RPE cell proliferation and regeneration. (A-J) *en face* wholemount images of unablpated (A-E) and ablated (F-J) larvae exposed to 2-hour EdU pulses immediately before fixation and staining for ZPR2 at various time points post-injury. (K) Cartoon depicting the 4 categories into which the RPE was divided, based on cellular pigmentation and intensity of ZPR2 expression: injury site cells peripheral RPE (pigmented, dimly ZPR2^+^), differentiated RPE (pigmented, ZPR2^+^), transition zone (lightly pigmented, ZPR2^+^ consisting of incompletely differentiated RPE extending into the injury site, and injury site (unpigmented, ZPR2^-^) (L) Quantification of the area of RPE comprised by each domain during regeneration reveals the degeneration of a large proportion of RPE rapidly after ablation, and that regeneration also rapidly occurs, with a transition zone appearing by 1dpi, and differentiated RPE reappearing in the periphery by 2dpi. The entire RPE is repopulated by differentiated RPE by 7dpi. (M-N) Transverse cryosections of unablated (M) and ablated (N) eyes at 1dpi. Magnified insets (M’,N’) reveal the presence of EdU^+^ cells in the RPE periphery in ablated retinae (N’ arrowhead). (O-R) Quantification of the density of EdU cells throughout regeneration in the peripheral RPE (O), Transition Zone (P), differentiated RPE (Q) and Injury Site (R) suggest that peripheral cells respond to injury by proliferating, that proliferation continues within newly-generated RPE and halts after regeneration is repopulated.

Next, we sought to obtain greater spatial and temporal resolution in our analysis of proliferative cells in the RPE layer. Therefore, larvae 1-7dpi were exposed to short 2-hour pulses of EdU and subsequently eyes were enucleated, stained for ZPR2/EdU, and imaged to acquire *en face* views of the entire RPE (Fig 11). To quantify the spatial dynamics of RPE cell ablation and regeneration, we divided the RPE of each eye into four regions based on cell location and two markers of differentiated RPE: pigmentation level and ZPR2 staining. The four RPE regions were delineated as follows: (1) peripheral RPE cells are pigmented and dimly ZPR2^+^, (2) differentiated RPE cells are highly pigmented and ZPR2^+^ (3) transition zone RPE cells are lightly pigmented but ZPR2^+^ and therefore likely consist of differentiating RPE extending into the injury site, and (4) the injury site, which contains no identifiable RPE cells, and which is often filled with aggregates of what are likely GFP^+^ and/or ZPR2^+^ debris (Fig 11K). Using these criteria to quantify RPE layer composition, our analysis confirmed that a large proportion of the RPE degenerates rapidly after ablation (Fig 11F and 11L). Strikingly, these analyses also revealed that differentiating RPE cells form a transition zone as soon as 1dpi (Fig 11G inset), and newly-formed differentiated RPE reappear in the periphery at 2dpi (Fig 11H and 11L). As regeneration proceeded, ZPR2^+^ transition zone cells always appeared in the periphery, stretching between the region of differentiated RPE and the central injury site. Furthermore, the proportion of the RPE encompassed by the transition zone at each time point correlated with the proportion of differentiated RPE cells added at the following time point, strongly suggesting these transitional RPE cells differentiate into regenerated RPE (Fig 11L). These analyses confirm earlier analysis showing that new RPE is added to the peripheral injury site, and that regeneration of a pigmented ZPR2^+^ RPE is completed by 7dpi.

Analysis of EdU^+^ cells revealed that there are more EdU^+^/ZPR2^+^ cells in the peripheral RPE of ablated larvae at 0.5dpi and 1dpi, and though this increase did not achieve significance (Fig 11O, p = 0.076 and p = 0.078, respectively), cryosections of 1dpi eyes showed peripheral EdU^+^ZPR2^+^ cells similar to those observed after BrdU exposure (compare Fig 11M and 11N to Fig 10G). During these early time points, EdU^+^/ZPR2^+^ cells were largely restricted to the peripheral retina, with only a few EdU^+^ cells appearing in the injury site (Fig 11R). During intermediate time points, when RPE cells reappear and the transition zone extends centrally, EdU^+^/ZPR2^+^ cells were present in both. As regeneration proceeded, the density of EdU^+^/ZPR2^+^ in regenerated RPE decreased, while increasing in the transition zone (Fig 11P and 11Q). By 4dpi, proliferative cells were largely restricted to the center of the eye (Fig 11I), and the majority of the remaining proliferative cells were located either in the injury site or the transition zone. Interestingly, the transition zone and regenerated RPE contained an even mix of EdU^+^ and EdU^+^/ZPR2^+^ cells, which may suggest that some differentiated RPE cells remain proliferative in this region and continue to generate new EdU^+^/ZPR2^-^ cells that later enter the transition zone and differentiate. As expected, proliferative cells were also observed in the CMZ, particularly during early time points (e.g. Fig 11A, 11F, 11M and 11N and Fig 10A–10H); however, there appeared to be fewer proliferative CMZ cells beginning at 2dpi. As part of our experimental paradigm, embryos were incubated in PTU until 5dpf, and therefore it is possible that PTU withdrawal elicits a proliferative response throughout the retina, or that CMZ proliferation may ordinarily decelerate starting at 7dpf. Since both ablated and unablated larvae have fewer proliferative CMZ cells at later time points, it is unlikely that this phenomenon is a critical factor influencing RPE regeneration. Taken together, these results strongly suggest that peripheral RPE respond to injury by proliferating, that proliferative RPE cells and/or their progeny move into the injury site, and that proliferation continues within newly-generated RPE cells adjacent to the injury site until the lesion is repopulated.

### Proliferative cells differentiate into regenerated RPE

**Fig 12:**
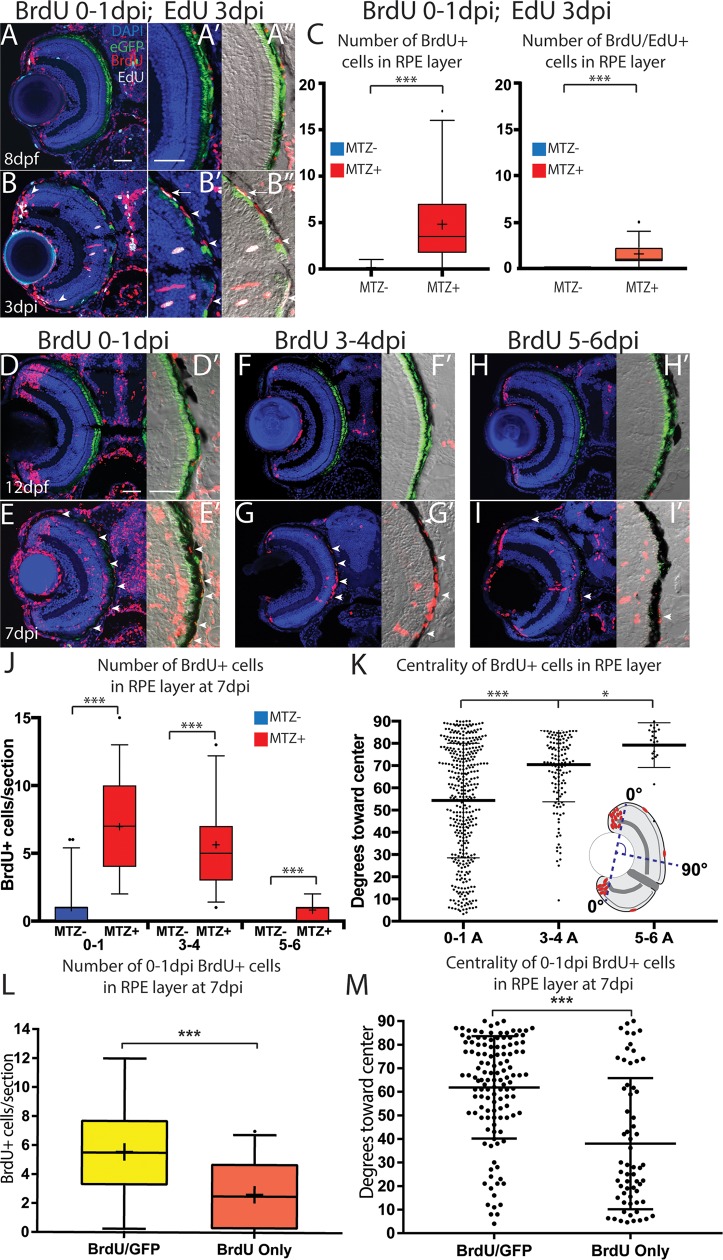
Proliferative RPE contributes to the regenerated RPE monolayer. (A-A”) Transverse sections from unablated larvae exposed to BrdU from 5-6dpf and pulsed with EdU for 2 hours before fixation at 8dpf. (B-B”) Transverse sections of ablated larvae exposed to BrdU from 0-1dpi and pulsed with EdU for 2 hours before fixation at 3dpi. (A’,B’) Magnified inset of BrdU/EdU. (A”,B”) Magnified inset of BrdU/EdU and DIC. Arrowheads in (B) highlight BrdU^+^ PRs that have integrated into the ONL. Arrow in (B’,B”) highlights a proliferative RPE cell, and arrowheads highlight unpigmented, previously-proliferative RPE-like cell in the injury site. (C) Quantification of BrdU/EdU^+^ and BrdU^+^ nuclei in the injury site. (D,E) Larvae exposed to BrdU 0-1dpi and fixed at 7dpi. (F-G) Larvae exposed to BrdU 3-4dpi and fixed at 7dpi. (H,I) Larvae exposed to BrdU 5-6dpi and fixed at 7dpi. (J) Quantification of the average number of BrdU^+^ cells per section. (K) Quantification of the location of individual BrdU^+^ cells relative to the center of the RPE. The line indicates the average location of BrdU^+^ cells, and the whiskers indicate standard deviation. (L,M) Quantification of BrdU^+^ cells that were labeled 0-1dpi within the RPE at 7dpi in ablated larvae. Analysis of eGFP^+^BrdU^+^ and GFP^-^BrdU^+^ cells in the RPE reveal that most BrdU cells in the RPE are eGFP^+^ at 7dpi. (C) Quantification of the location of individual BrdU^+^ cells relative to the center of the RPE indicates that eGFP^+^BrdU^+^ cells tend to be located toward the center and eGFP^-^BrdU^+^ localize toward the periphery. Mann-Whitney U Test, * p<0.05, ** p<0.005, *** p<0.0005. Scale = 100μm. Dorsal is up and distal is left. Scale bar = 40um.

We were interested in the EdU^+^/Zpr2^+^ differentiated RPE and the possibility that they continue to proliferate after injury. Thus, to determine whether early-proliferative cells enter the injury site and continue proliferating, we pulsed ablated larvae with BrdU between 0-1dpi, and with EdU at 3dpi before fixation and analysis (Fig 12A and 12B). Transverse sections revealed a significant increase of BrdU^+^/EdU^+^ cells and BrdU^+^ cells within the RPE of ablated fish (Fig 12C, p<0.0001). Interestingly, BrdU^+^/EdU^+^ cells often appeared at the interface between pigmented RPE and the unpigmented injury site, and some appeared to be pigmented (Fig 12B”). We next sought to determine whether early-proliferative cells ultimately integrate into the regenerated RPE. To do this, we exposed ablated larvae to BrdU between 0-1dpi and fixed them at 7dpi for analysis (Fig 12D and 12E). Transverse sections revealed a significant increase of BrdU^+^ cells within the RPE (Fig 12E and 12J, p<0.0001). These data suggest that early-proliferative cells enter the injury site at the leading edge of the regenerating RPE layer and either continue proliferating or give rise to proliferative cells there. Were this the case, we hypothesized that early-proliferative cells would integrate into both the peripheral and central RPE layer, while later-proliferative cells would form RPE only within the central RPE. To assess this, we pulsed ablated larvae with BrdU at 3-4dpi or 5-6dpi before fixing at 7dpi (Fig 12F–12I). BrdU^+^ cells were distributed throughout the RPE after a 0-1dpi pulse, but became more restricted to the central RPE after 3-4dpi and 5-6dpi pulses (Fig 12K). This analysis demonstrated that proliferative cells at early time points were distributed throughout the RPE, while later-proliferative cells were restricted to the central RPE. Finally, to determine whether early-labeled proliferative cells ultimately differentiate into RPE by 7dpi, we quantified the number and centrality of BrdU^+^/eGFP^+^ cells in 7dpi larvae that had been pulsed with BrdU between 0-1dpi (Fig 12L and 12M). Our analysis revealed that significantly more BrdU^+^ cells in the RPE were eGFP^+^ than eGFP^-^ (Fig 12L, p = 0.0005), and that BrdU^+^/eGFP^+^ cells preferentially integrated toward the center (Fig 12M, p<0.0001). In summary, these data indicate that early-proliferating cells in the RPE layer ultimately differentiate into regenerated RPE, and strongly suggest that these proliferative cells are located in the periphery and that they or their progeny migrate into the injury site.

### RPE regeneration is impaired using a chemical inhibitor of Wnt signaling

**Fig 13:**
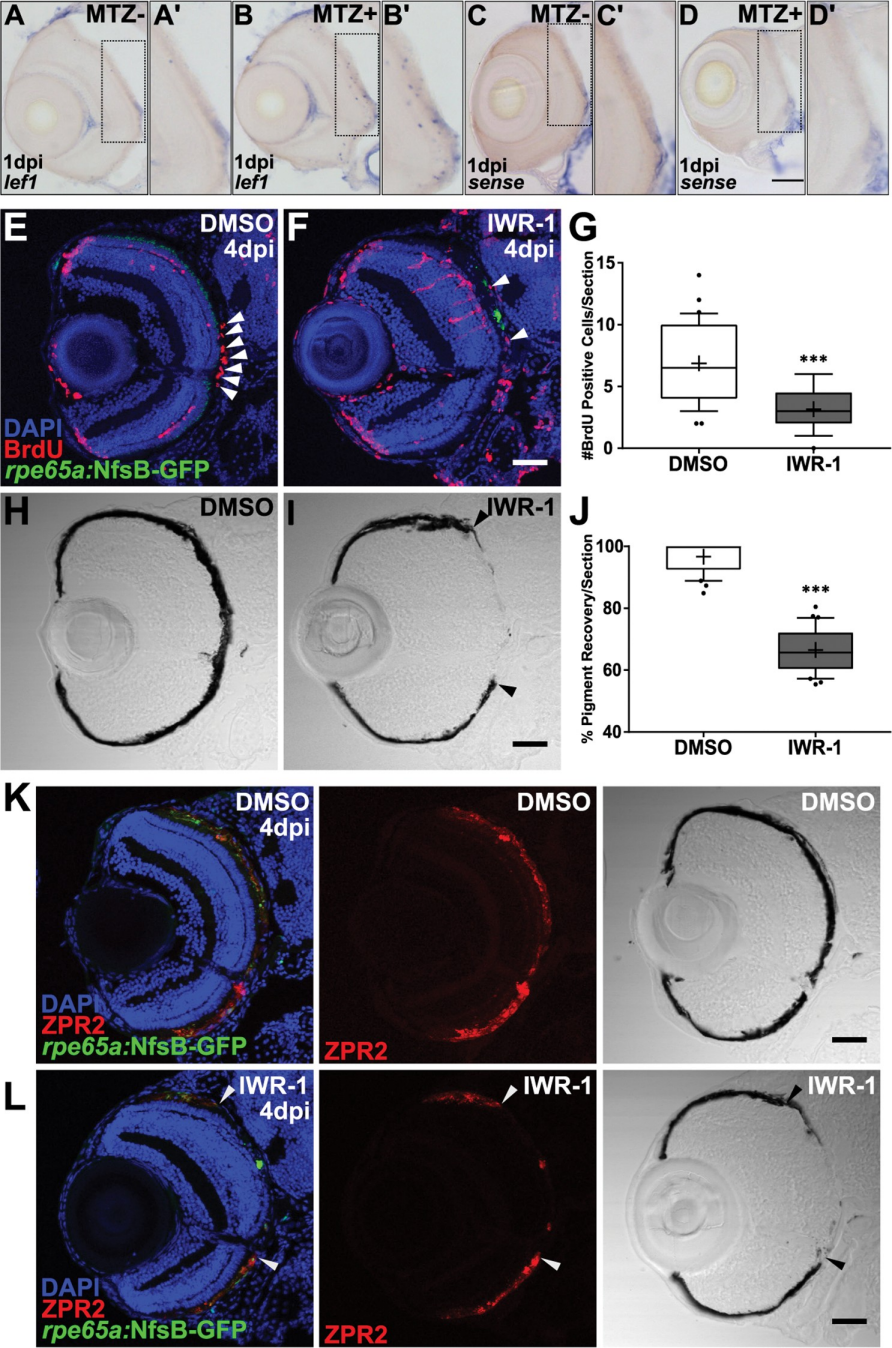
Pharmacological inhibition using IWR-1 impairs RPE regeneration. (A-D) Transverse sections of *lef1* or sense RNA expression in unablated 6dpf (MTZ-) and ablated 1dpi (MTZ+) larvae. *lef1* is detected in and around the RPE in MTZ+ (B’) but not MTZ- larvae (A’). *lef1*: *n>5*; *lef1 sense*: *n = 4*. (E-J) Transverse sections of 4dpi ablated DMSO- (E,H; *n = 10*) and 15μM IWR-1-treated (F,I; *n = 11*) larvae exposed to a 24-hour pulse of BrdU from 3-4dpi. (E,F) Green = eGFP, blue = DNA, red = BrdU; *white arrowheads* highlight BrdU+ cells in the RPE. (G) Quantification of BrdU+ cells/section reveals that IWR-1 treatment significantly decreases the number of proliferative cells in the RPE at 4dpi (Student’s unpaired t-test, *** p<0.0001). Brightfield images (H,I) and quantification of percent RPE recovery/section (J) shows a significant delay in recovery of a pigmented monolayer in IWR-1 treated larvae (Student’s unpaired t-test, *** p<0.0001). (I) *Black arrowheads* indicate the central-most edge of the regenerating RPE. Transverse sections of 4dpi ablated DMSO- (K; *n = 6*) and 15μM IWR-1-treated (L; *n = 5*) larvae stained for ZPR2 *(red)*. Green = eGFP and blue = nuclei. ZPR2 staining overlaps with a thick, heavily pigmented regenerated RPE monolayer. (L) *Arrowheads* indicate the central-most edge of the regenerating RPE. In IWR-1-treated larvae, ZPR2 staining is not observed central of the rim of pigment indicating a lapse in RPE regeneration, not a pigmentation deficiency. Dorsal is up and distal is left. Scale bars = 40μm.

Our results thus far provide the first demonstration in any model system that RPE can endogenously regenerate after widespread injury. Next, we wanted to leverage this *in vivo* system to begin to identify the molecular underpinnings of the regenerative response. Previous studies have identified Wnt signaling as a regulator of tissue regeneration in multiple contexts [63–69], including the retina [70–72], and possibly RPE [73]. Thus, we examined Wnt signaling to begin to gain mechanistic insight into the molecular mechanisms underlying RPE regeneration. To assess Wnt pathway activity after RPE ablation, we examined expression of the Wnt target gene, *lef1* [69,74]. *lef1* was upregulated in ablated larvae at 1dpi (Fig 13B and 13B’), but not in unablated siblings (Fig 13A and 13A’) or in sense controls (Fig 13C, 13C’,13D and 13D’). Closer analysis of *lef1* expression in ablated eyes revealed transcripts distributed in and adjacent to the RPE layer (Fig 13B’), suggesting the Wnt pathway is activated post-ablation. We next utilized IWR-1, which stabilizes Axin2 and promotes destruction of ß-catenin [75], to determine if disrupting Wnt pathway components impedes RPE regeneration. Larvae were pre-treated 24 hours prior to ablation (4dpf/-1dpi) with 15μM IWR-1 or with a vehicle control (0.06% DMSO) and kept in drug or vehicle until fixation at 4dpi (the time at which peak proliferation is observed in the RPE layer (Fig 10U)). Quantification of BrdU^+^ cells/section revealed a significant decrease in proliferation in IWR-1-treated RPE when compared to controls (Fig 13E–13G, p<0.0001). Further, there was a noticeable lapse in recovery of a pigmented monolayer in IWR-1-treated larvae (Fig 13I, *arrowheads*) relative to DMSO controls (Fig 13H). ZPR2 staining overlapped with pigmented RPE in both ablated DMSO- (Fig 13K) and IWR-1-treated (Fig 13L) larvae, indicating the lapse in pigment recovery was not simply a pigmentation deficiency, but rather a failure of the RPE to regenerate. Quantification of percent RPE recovery indeed showed a significant decrease in the IWR-1-treated larvae (Fig 13J, p<0.0001). These data suggest that components of the Wnt signaling pathway may be involved in RPE regeneration.

## Discussion

The stimulation of endogenous RPE regeneration is an appealing possibility for treating degenerative RPE diseases. However, the development of such a therapy is constrained by the paucity of data regarding the cellular and molecular underpinnings of regeneration. While the mammalian RPE possesses a latent proliferative ability, the process by which RPE cells respond to damage by proliferating and regenerating a functional monolayer, remains largely unknown.

The development of an animal model of RPE regeneration following specific and widespread RPE damage is a critical first step towards elucidating the regenerative process. Here, we developed a zebrafish model to ablate mature RPE and assess its regenerative capacity. In this model, ablation of a large contiguous stretch of RPE led to apoptosis and degeneration of the majority of mature RPE, which was rapidly followed by BM and PR degeneration and loss of visual function. In comparison, most RPE injury/regeneration models create small lesions using non cell-specific injury techniques (e.g. debridement or laser photocoagulation; [32,76,77]), or ablate a diffuse subpopulation of RPE cells via sodium iodate [78–82]. In mouse, a genetic RPE ablation system expressing diphtheria toxin in a subpopulation of RPE did not cause BM degradation or RPE proliferation [54]. Indeed, many RPE injury models preserve an intact BM or spare large regions of RPE (e.g. [54,83]). In contrast, our zebrafish model creates RPE and photoreceptor degeneration, which more closely resembles defects observed in late-stage AMD, wherein RPE dysfunction and degeneration precedes PR loss [12,84] [85,86], and thus may represent a more clinically-relevant starting point than other extant models for studying RPE regeneration.

Remarkably, we found that zebrafish are capable of regenerating after such a severe injury: within 7-14dpi in larvae, and within 1 month in adults. To our knowledge, these data provide the first evidence of RPE regeneration after widespread injury in any model system. Mammals largely fail to regenerate a functional RPE monolayer following injury [25,26]. One exception to this is in “super healer” MRL/MpJ mice, which regenerate the RPE within ~30 days after administration of mild doses of sodium iodate that elicit degeneration of the central RPE [51]. Beyond this example, mammalian RPE are incapable of regenerating after severe injuries (e.g. 27–29, 31). Our zebrafish RPE ablation model differs significantly from *Xenopus* [87], newt [88,89], and embryonic chick [90–92] retinectomy models wherein the entire retina is surgically removed and subsequently regenerates from remaining RPE tissue that that transdifferentiates, proliferates, and regenerates retinal tissue. Studies in these models have focused on the RPE-to-retina transdifferentiation process, and RPE-specific regeneration remains unexplored.

**Fig 14:**
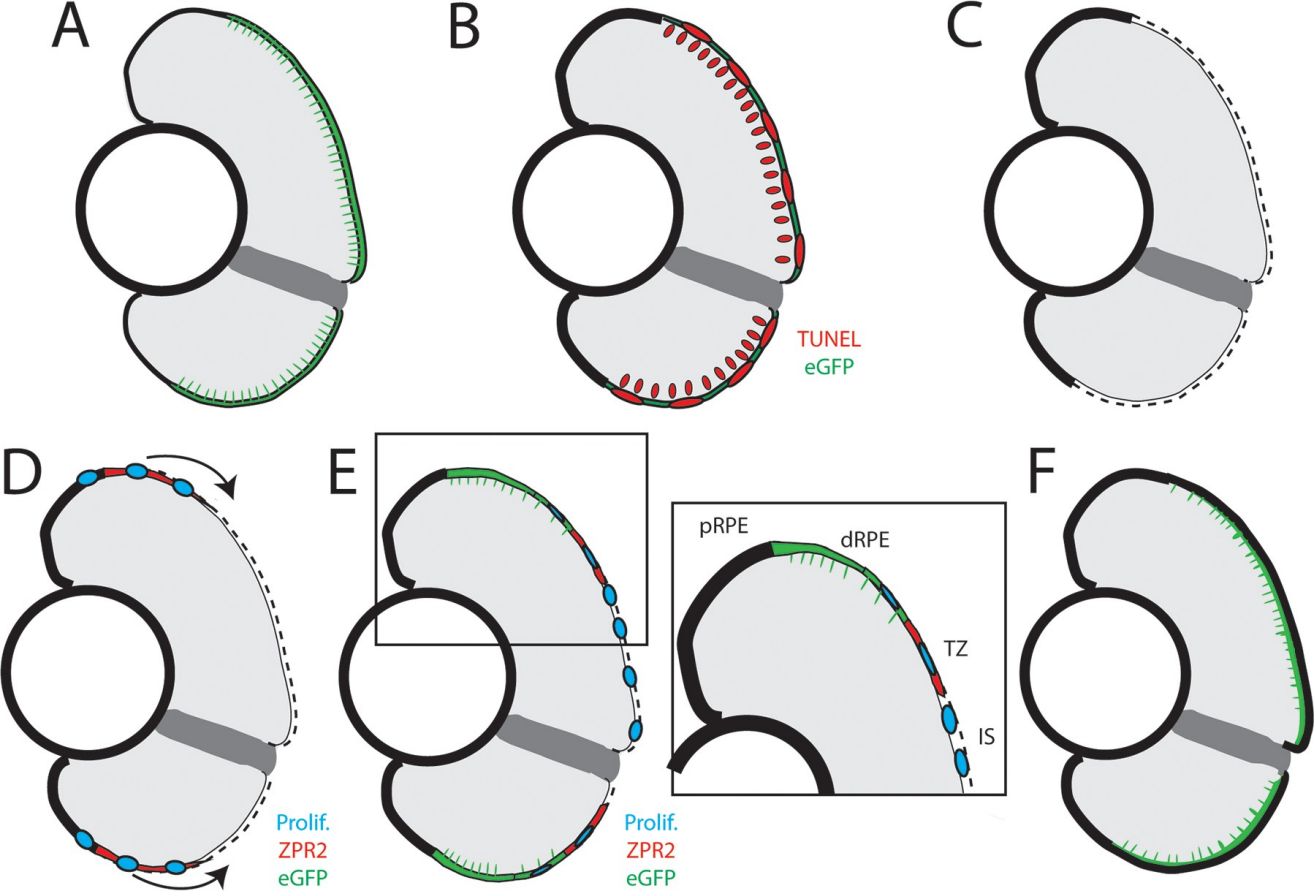
Model of RPE regeneration in larval zebrafish. (A) nfsB-eGFP is specifically expressed in mature RPE in the central two-thirds of the eye. (B) Application of MTZ leads to apoptosis (red) of RPE and PRs. (C) RPE ablation leads to degeneration of PRs and Bruch’s Membrane (dotted line). (D) Unablated RPE in the periphery begin to proliferate and extend into the injury site (blue). (E) As regenerated eGFP^+^ RPE appear in the periphery, the RPE can be divided into 4 zones: peripheral RPE, differentiated RPE, transition zone, and injury site. (E, inset) Regenerated differentiated RPE appears in the periphery proximal to the unablated peripheral RPE, and contains proliferative cells adjacent to the transition zone. The transition zone consists of still-differentiating RPE cells and proliferative cells. The injury site is comprised of unpigmented proliferative cells that do not express any RPE differentiation markers. (F) Regeneration of a functional RPE layer and Bruch’s Membrane is complete by 14dpi.

We present data here demonstrating that both larval and adult zebrafish possess the capacity to regenerate their RPE. However, due to the technical advantages of using larvae in studying regeneration (i.e. rapid regeneration, large sample sizes, feasibility of *in vivo* imaging, utility of the available genetic toolkit, and the ability to perform high-throughput drug screens) we mainly focused on characterizing RPE regeneration during larval stages. In larvae, regenerated RPE appeared at the periphery of the injury site at 2dpi, and the entire lesion was repopulated with differentiated RPE cells within 1 week. Our data support the following model of larval RPE regeneration (Fig 14): injury-adjacent RPE expand into the injury site, where they encounter degraded BM and proliferate to form daughters that enter the injury site and differentiate into RPE. RPE commonly expand to fill territory vacated by lost RPE [24,54,93], and contact with a degenerated BM induces RPE proliferation in many contexts [24,30,35,94–96]. Supporting this, we found that early-dividing cells (0-1dpi) often appear in the RPE periphery, localize to the injury site during peak phases of regeneration, and ultimately form RPE that integrate into the regenerated RPE monolayer. Wholemount analyses indicated that proliferative cells appear in the peripheral RPE soon after injury, and proliferative cells differentiate into RPE in distinct zones: (1) newly differentiated injury-adjacent RPE, (2) a transition zone, containing actively differentiating RPE cells, and (3) the injury site, which contains cellular debris as well as some proliferative cells that do not yet express RPE markers. Further experiments are necessary to determine whether all injury-adjacent RPE are capable of proliferating in response to injury, or if proliferation occurs within a subpopulation. Several lines of evidence suggest the latter possibility, and highlight the important role played by peripheral RPE: in mouse, a subpopulation of mature RPE in the periphery remain in the cell cycle and respond to microscopic photocoagulation injuries by proliferating at a higher rate than central RPE [32,97], while experiments in pig have shown that peripheral RPE respond to debridement of central RPE by proliferating [98]. Indeed, preservation of the peripheral RPE is also a prerequisite for successful RPE regeneration in the MRL/MpJ mouse model, which fails to regenerate RPE when high doses of sodium iodate cause degeneration of both central and peripheral RPE [51,99]. Finally, the discovery of a subpopulation of RPE stem cells [36] suggests that an endogenous regeneration-capable population of RPE could exist in the human eye, and these might be analogous to injury responsive cells in zebrafish.

While we present strong evidence supporting a model in which regenerated RPE derives from injury-adjacent RPE, we cannot definitively establish the source of regenerated RPE without performing lineage tracing. In response to retinal injury, Muller glia proliferate and generate MGPCs that differentiate into new neurons [37,58]. MGPCs are present at identical time points and in regions adjacent to ablated RPE. While it is possible that MGPCs transdifferentiate into RPE, this ability is not supported by any published studies. Another possible source for regenerated RPE is the CMZ, which generates new neurons throughout the life of the animal [100–102]. It was recently shown that *rx2*^+^ stem cells in the CMZ generate both RPE and retinal neurons [103]. Thus, *rx2*^+^ cells in the CMZ could potentially respond to RPE injury by generating RPE in the periphery that migrate and proliferate within the injury site. Attempts at lineage tracing to date have been unsuccessful and therefore, it will be necessary to develop new genetic tools to unambiguously identify the source of regenerated RPE.

We were surprised by the recovery of the OKR at 3dpi, well before the complete regeneration of the RPE layer (Fig 4). The threshold number of PRs required for a positive OKR in zebrafish is unknown. However, since the OKR is elicited by a large-field stimulus, activating PRs throughout the eye, this early recovery could be driven by CMZ-derived PRs that integrate into the peripheral retina by 3dpi. Consistent with this hypothesis, we observed CMZ-derived BrdU^+^ PRs in the retinal periphery at 3dpi (Fig 12B). It is also possible that recovered PRs adjacent to newly regenerated RPE in the peripheral injury site contribute to the OKR before regeneration of the central injury is complete. Alternatively, it is possible that a sub-population of central photoreceptors is functionally compromised, but survives ablation of the RPE, and these recover function by 3dpi. More work will be required to elucidate which of these processes might contribute to the rapid recovery of visual function at 3dpi.

Wnt signaling plays a known role in multiple regenerative contexts [63–69], including in the eye [70–72]. We show here that the Wnt pathway is activated in a subset of cells after RPE injury and that chemical inhibition using IWR-1 impairs regeneration. While we have not identified the *lef1*-expressing cell type, two possible sources are: 1) apoptotic cells in the injury site and 2) cells of the immune system. First, there are notable similarities between *lef1* expression and TUNEL staining in the ONL and the RPE, and we detect *lef1* expression (Fig 13B and 13B’) at the same time that TUNEL^+^ cell numbers peak in ablated larvae (Fig 2D–2F). A previous study in *Hydra* showed that apoptotic cells are a source of Wnt3, and that this is required for head regeneration [104]. The immune system has also been shown to play a critical role in influencing the regenerative response [105–107] and Wnt signaling regulates the inflammatory properties of immune cells. Wnt signaling is also important for RPE development *in vivo* [108–110], and it is possible that Wnt activation is important at multiple time points during RPE regeneration for initiation and to stimulate progenitor proliferation and/or differentiation. Further work is required to distinguish between cells in which Wnt is activated and regenerating RPE, and ultimately, these experiments may determine how the Wnt signaling pathway modulates RPE regeneration.

Finally, as noted above, we observed signs of BM degeneration in the central injury site following ablation. That RPE ablation leads to BM breakdown may provide insight into the mechanisms underlying the initiation of CNV at the onset of exudative AMD. During CNV, choroidal endothelial cells penetrate the BM and grow into the subretinal space; whether this process is initiated by the degeneration of the choriocapillaris or the RPE remains controversial [105,111,112]. Our results suggest that the RPE is required for BM maintenance. A logical next step would be to determine if choroidal vasculature invades the subretinal space following degeneration, as this would provide evidence that RPE degeneration is causative of CNV. Additionally, we show that zebrafish RPE are capable of repairing the BM during regeneration. In human, the BM undergoes a series of changes during aging that are thought to underlie AMD pathogenesis and inhibition of RPE function, changes that are thought to underlie the barrier to development of successful RPE transplant therapies [113,114]. The mechanisms underlying BM repair in zebrafish may provide critical insights into improving transplant survival and reintegration in humans.

## Materials and methods

### Fish maintenance and husbandry

Zebrafish were maintained at 28.5°C on a 14-hour light/10 hour dark cycle. Embryos were obtained from the natural spawning of transgenic or wild-type parents in pairwise crosses. According to established protocols [45], embryos were collected and raised at 28.5°C in the dark until they reached appropriate ages for experimentation. *rpe65a*:nfsB-eGFP was propagated by outcrossing to AB-strain wild-type fish. All animals were treated in accordance with provisions established by the University of Texas at Austin and University of Pittsburgh School of Medicine Institutional Animal Care and Use Committees.

### Tol2 transgenesis

To establish the *rpe65a*:nfsB-eGFP transgenic, 0.8KB immediately upstream of the coding sequence of rpe65a was cloned into a p5E entry vector, and this was placed upstream of nfsB-eGFP using the Multisite Gateway Cloning system (Thermo Fisher, Waltham, MA) within a backbone vector enabling Tol2-mediated transgenesis. This construct was then inserted into the genome using Tol2 recombinase [115].

### RPE ablation

Embryos derived from *rpe65a*:nfsB-eGFP x AB crosses were maintained in PTU-containing (Sigma-Aldrich, St. Louis, MO) fish water between 1-5dpf, and dechorionated at 3dpf. At 5dpf, larvae were exposed to 10mM metronidazole (MTZ; Sigma-Aldrich), dissolved in fish water, for 24 hours in the dark. After treatment, larvae were removed from MTZ and allowed to recover in fish water for 48 hours. At this point, severely ablated embryos were selected based upon the disruption of eGFP signal in the RPE and lack RPE pigmentation. Adult zebrafish (age 11 months) were ablated using the same parameters as for larvae: 24-hour 10mM MTZ treatment in a light-blocking incubator. Post-MTZ treatment, adults were returned to a 14-hour light/10 hour dark cycle and allowed to recover for 48 hours. Post-recovery, all ablated adults and larvae were placed on the recirculating water system (Aquaneering, San Diego, CA).

### Quantification of overall RPE pigmentation

Larvae were euthanized at 2, 4, or 7dpi and fixed in 4% paraformaldehyde (PFA) (Thermo Fisher) overnight. Eyes were enucleated and the lens was removed before mounting in 3% methylcellulose. Images were acquired at a 112X magnification on a Zeiss (Oberkochen, Germany) Axio Zoom.V16 microscope. MetaMorph Image Analysis Software (Molecular Devices, San Jose, CA) was used to normalize the background of each image, delineate a region of interest (ROI) around each eye, and measure the average intensity (total intensity within the ROI divided by the area of the ROI). To quantify pigment (gray intensity) rather than white intensity, values were inverted by subtracting the generated intensity value from 65,536 (the number of gray levels in a 16-bit image).

### BrdU and EdU exposures

For BrdU labeling, larvae were incubated in system water containing 10mM BrdU (Sigma-Aldrich) for 24 hours at various time points. After incubation, larvae were either immediately fixed for analysis or rinsed with fresh system water and then allowed to recover until 7dpi. For EdU labeling, larvae were incubated in system water containing 500μM EdU (Thermo Fisher, #C10338) for 2 hours immediately prior to fixation with 4% PFA in PBS.

### Immunohistochemistry

Larvae were euthanized with Tricaine (Spectrum Chemical, New Brunswick, NJ) before fixation in 4% PFA overnight at 4°C, and adults were euthanized with Tricaine before removal of the eyes. Larvae and adult eyes were then sucrose-protected and embedded in tissue-freezing medium (TBS, Inc., Waltham, MA) before being sectioned at 14μm (larvae) or 16μM (adults) on a Leica CM1850 cryostat. Sections were rehydrated in 1xPBS for 5 minutes, and blocked in 5% normal goat serum in PBS for 2 hours at room temperature. For BrdU imaging, sections were treated with 4N HCl for 8 minutes at 37 degrees for antigen retrieval. Sections were counterstained with 1:500 DAPI (Life Technologies, Waltham, MA) for 9 minutes at room temperature, washed 3X with PBS, and mounted with Vectashield (Vector Laboratories, Burlingame, CA). Images were obtained with a 40X objective on an Olympus (Tokyo, Japan) FV1200 confocal microscope. Antibodies used in this study include BrdU (Abcam, Cambridge UK, ab6326), ZPR2 (ZIRC, Eugene, OR), ZPR1 (ZIRC), Phalloidin (Thermo Fisher, A22284, 1:33 dilution). TUNEL (Roche, Mannheim Germany, #12145792910) and Click-It EdU staining (Thermo Fisher, C10618) were performed according to manufacturer’s instructions.

### Staining and dissection for wholemount analysis of the RPE

After fixation, larvae were prepared using a protocol adapted from [116]: larvae were rinsed in PBST and water before permeabilization using acetone (-20°C for 12 minutes), rinsing in PBST, 1mg/mL Collagenase (Sigma-Aldrich) (30 minutes at RT), and 2mg/mL Proteinase K (Thermo Fisher, cat#BP1700) (30 minutes at RT). Larvae were refixed in 4% PFA (20 minutes at RT), rinsed in PBST, blocked using PBDTX (PBS + 1% bovine serum albumin, 1% DMSO and 0.1% Triton X-100), and stained overnight in PBDTX at 4°C with primary antibodies listed above at 1:250. Larvae were rinsed PBDTX on a shaker at RT (4X for 15 minutes), incubated with secondary antibodies (1:250), rinsed with PBDTX again (4X for 15 minutes) and counterstained with DAPI (1:200 in PBS). Chemically etched Tungsten needles [117] were used to dissect the eye and remove the choroid, and RPE flatmounts were mounted on Superfrost slides in PBS immediately prior to imaging.

### *In situ* hybridization

Whole mount *in situ* hybridization was performed as described [118] using a previously reported DIG-labeled RNA probe for *lef1* [119]. Post-labeling, larvae were fixed overnight in 4% PFA at 4°C then sucrose-protected, embedded, sectioned (12μm), and mounted using DPX Mounting Medium (Electron Microscopy Sciences, Hatfield, PA). Images were obtained with a 40X objective on a Zeiss Observer.Z1 inverted microscope.

### Transmission electron microscopy

Embryos were fixed in fresh 4% glutaraldehyde, 2% PFA overnight at room temperature, stained in 2% osmium tetroxide (OsO_4_) and 2% potassium ferrocyanide and 2% uranyl acetate, and microwave-embedded with a modified reduced-viscosity Spurr-Quetol-651 resin using a BDMA accelerator (Electron Microscopy Sciences) via a 30, 50, 75, 100, and 100% resin/acetone infiltration series [120]. Samples were sectioned using a Leica Ultracut UC7 ultramicrotome at a thickness of 70nm, and imaged on a FEI Tecnai transmission electron microscope.

### Measurement of BM thickness

For quantification of BM thickness, three transverse sections of the eye including the optic nerve were taken from the central RPE injury site in n = 3 zebrafish. The central RPE was defined as the region between the optic nerve head and the intersection of the dorsal eye and brain, as this correlated both with the area of highest transgene expression in unablated larvae, and degeneration in ablated larvae. In each section, three images of the BM were collected at 20,500X magnification in each image, and in each image, 3 measurements of the BM were taken by drawing lines perpendicular to the retina connecting the retinal and choroidal surface of the BM. In summary, 3 lines were drawn per image, 3 images taken per section, and 3 sections taken per animal for a total of 27 BM measurements per larva.

### OKR assays

Larvae were immobilized in 3% methylcellulose, oriented dorsal up and exposed to a full field rotating stimulus projected onto a screen (NEC, Itasca, IL) that encompassed 180 degrees of the stimulated eye’s field of vision. Responses were captured using infrared light (880nm; Spectrum, Montague, MI) through a Flea3 Camera (Point Grey Research, Richmond, BC, Canada) mounted on a dissecting microscope (Leica Microsystems, Wetzlar, Germany). Videos were recorded by FlyCapture software (FLIR, Richmond, BC, Canada) and quantified using custom MATLAB scripts [50].

### OCT imaging

Larvae were immobilized dorsal up in 3% methylcellulose and imaged with Optical Coherence Tomography (OCT) (~840nm; Leica Bioptigen R2210 Spectral Domain Ophthalmic Imaging System). After imaging, larvae were rinsed and transferred into petri dishes to recover. OCT scans were analyzed using Bioptigen software (InVivoVue; Bioptigen, Research Triangle Park, NC), FIJI, and MetaMorph.

### Wnt pathway manipulation

Larvae were treated with 15μM IWR-1-endo (Sigma-Aldrich) or 0.06% dimethyl sulfoxide (DMSO; Thermo Fisher) as a vehicle control from 4dpf/-1dpi until 9dpf/4dpi with daily replenishing of water and treatment. To assay proliferating cells, 10mM BrdU (Sigma-Aldrich) was added for 24 hours prior to fixation at 9dpf/4dpi. BrdU immunohistochemistry was performed as described.

### Experimental design and statistics

For quantification of apoptosis, TUNEL^+^ cells within the RPE layer and ONL were recorded in unablated and ablated larvae at 3, 6, 12, 18, 24 and 48hpi. For quantification of BrdU^+^ nuclei in the RPE layer, BrdU^+^ nuclei contained within the RPE monolayer or presumptive RPE space between the retina and choroid were counted. To quantify the number of BrdU^+^ nuclei in the central retina, a reference line was drawn between the proximal-most BrdU^+^ cell originating from the CMZ in the dorsal and ventral retina. All BrdU^+^ cells in all layers of the retina proximal to this line were then counted. To quantify the centrality of BrdU^+^ cells, an additional perpendicular line bisecting the line demarcating CMZ-generated BrdU^+^ cells was drawn. BrdU^+^ cells in the RPE were counted using criteria detailed above, and the angle of each cell relative to these lines (0° ≤ x ≤ 90°) was calculated using FIJI software [121].

For quantification of RPE following OCT imaging, three transverse images of the retina were captured per fish at each time point (MTZ^-^ n = 10, MTZ^+^ n = 9). Using MetaMorph, a line was drawn on the RPE beginning just dorsal of the optic nerve and terminating at the dorsal periphery, and a linescan of pixel intensity was taken from this line. The average RPE pixel intensity from the optic nerve to the periphery was graphed using GraphPad Prism 7.04 for Windows (Microsoft, Redmond, WA) (error bars = SEM). To quantify positional differences, each graph was divided into quintiles and the area under the curve was calculated for each quintile and compared using the Student’s t test.

For quantification of adult RPE recovery, central sections from adult eyes (MTZ-, 3dpi, 7dpi, 35dpi n = 4, 14dpi n = 3) were obtained and the edges of the injury site were designated based on contiguous-expressed eGFP^+^/ZPR2^+^ (e.g. *arrowheads* in Fig 9B–9E). Measurements for: 1) intact RPE (contiguous eGFP^+^/ZPR2^+^ expression) and 2) degenerated RPE were made along the dorsal and ventral retinae using the line segment tool in FIJI (ImageJ). Measurements were made along the basal side of the RPE from the distal tip to the junction of the optic nerve head (ONH). Width of the ONH was omitted from these measurements to avoid variability between adults. Dorsal and ventral measurements were summed and adult RPE regeneration was represented as percent eGFP^+^/ZPR2^+^ RPE.

Normality of datasets was assessed by the D’Agostino-Pearson Omnibus test. When analyzing non-normal datasets, Mann-Whitney U test was utilized to determine the significance of differences between unablated and ablated larvae. In normally-distributed datasets, Student’s t-test was utilized. These analyses were performed by GraphPad Prism 7.0c Software for Mac OS (La Jolla, CA, www.graphpad.com) and Microsoft Excel 14.7.3 (Microsoft). All statistical analyses are included in S1 Table.

## Supporting information

S1 Fig Wholemount analysis of RPE pigmentation. (A-F) Representative images of wholemounted eyes from unablated (A-C) and ablated larvae (D-F) at 2,4, and 7dpi. (G) Quantification of the mean gray intensity per eye reveals significant depigmentation in ablated larvae at 2dpi, and that pigmentation significantly improves between 2dpi and 4dpi, though overall pigmentation remains significantly reduced compared to unablated controls at 7dpi (Welsh’s t test, * P<0.05, **p<0.005, ***p<0.0005).
(TIF)

S2 Fig Analysis of TUNEL^+^ cells in the ablated eye. (A-L) Transverse cryosections stained for TUNEL (red). (A,C,E,G,I,K) Unablated and (B,D,F,H,J,L) ablated eyes at various time points following ablation. While the ONL appears to be unchanged at 3hpi, slight disruptions in ablated RPE morphology are detectible: apical microvilli become shortened compared to control, and the occasional TUNEL+ nucleus appears in the RPE layer (B). By 6hpi, degeneration of the eGFP^+^ apical microvilli and cell bodies becomes notable throughout the injury site, and nuclear organization in the ONL begins to degenerate (D). By 18hpi, eGFP signal begins to accumulate in blebs, leaving regions devoid of eGFP^+^ cells, and TUNEL signal appears throughout the RPE and ONL (H). Degeneration of the central injury site is complete by 48hpi, and TUNEL signal is reduced (L).
(TIF)

S3 Fig Metronidazole treatment does not cause ONL or RPE apoptosis in nontransgenic larvae. (A-D) Transverse cryosections stained for TUNEL (red). No TUNEL^+^ cells were detected in nontransgenic larvae (A,C) treated with and without MTZ. (E,F) Quantification of TUNEL^+^ cells/section in the ONL (E) and RPE (F). While ONL death appeared to be elevated in unablated *rpe65a*:nfsB-eGFP^+^ larvae, the increase was not significant (p = 0.1848), and no significant elevation of TUNEL^+^ cells was detected in the RPE (Mann-Whitney U test, *P<0.05, **p<0.005, ***p<0.0005). Scale bar = 40μm.
(TIF)

S4 Fig ZPR1^+^ PRs adjacent to regenerated RPE in the retinal periphery appear normal. Transverse cryosections of unablated larvae (A,G) and ablated larvae (D,J) stained for ZPR2. Green = GFP, blue = nuclei, red = ZPR2. Magnified insets of central (B,E) and peripheral (E,F) RPE. (E-E”) At 3dpi, the ZPR1^+^ signal in PRs within the central injury site becomes less organized and reveals degeneration of outer segment tips. (F-F”) In the periphery, dim GFP signal marks the presence of regenerated RPE, and underlying ZPR1^+^ cones display morphologies similar to unablated controls. ZPR1^+^ cones closer to the periphery express normal levels of ZPR1 and identifiable outer segment tips, while those closer to the injury site display increasingly disorganized morphology and lack contiguous outer segment tips. (K-K” At 4dpi, ZPR1 signal continues to be degraded in the central injury site. (L-L”) In the periphery, where GFP^+^ RPE tissue has regenerated, ZPR1 staining is similar to unablated controls (I-I”), identifying organized cell bodies, outer segments, and outer segment tips. Scale bar = 40μm.
(TIF)

S1 Video A video depicting each optical slice of an unablated 6dpf (1dpi) larvae. The frames analyzed (62–64) are marked with arrows pointing at the upper and lower boundaries of the analyzed region.
(AVI)

S2 Video A video depicting each optical slice of an ablated 1dpi larvae. The frames analyzed (56–58) are marked with arrows pointing at the upper and lower boundaries of the analyzed region.
(AVI)

S3 Video A video depicting each optical slice of an unablated 8dpf (3dpi) larvae. The frames analyzed (59–61) are marked with arrows pointing at the upper and lower boundaries of the analyzed region.
(AVI)

S4 Video A video depicting each optical slice of an ablated 3dpi larvae. The frames analyzed (44–46) are marked with arrows pointing at the upper and lower boundaries of the analyzed region.
(AVI)

S5 Video A video depicting each optical slice of an unablated 10dpf (5dpi) larvae. The frames analyzed (45–47) are marked with arrows pointing at the upper and lower boundaries of the analyzed region.
(AVI)

S6 Video A video depicting each optical slice of an ablated 5dpi larvae. The frames analyzed (48–50) are marked with arrows pointing at the upper and lower boundaries of the analyzed region.
(AVI)

S1 Table Statistics. Table of all statistical analyses, n’s and p-values. Statistics are arranged by Figure and experiment.
(XLSX)
