# Supplementary figures and images for: Regeneration of the zebrafish retinal pigment epithelium after widespread genetic ablation
Source: PLoS Genet. 2019 Jan 29;15(1):e1007939. doi: 10.1371/journal.pgen.1007939 (PMC6368336; doi:10.1371/journal.pgen.1007939)

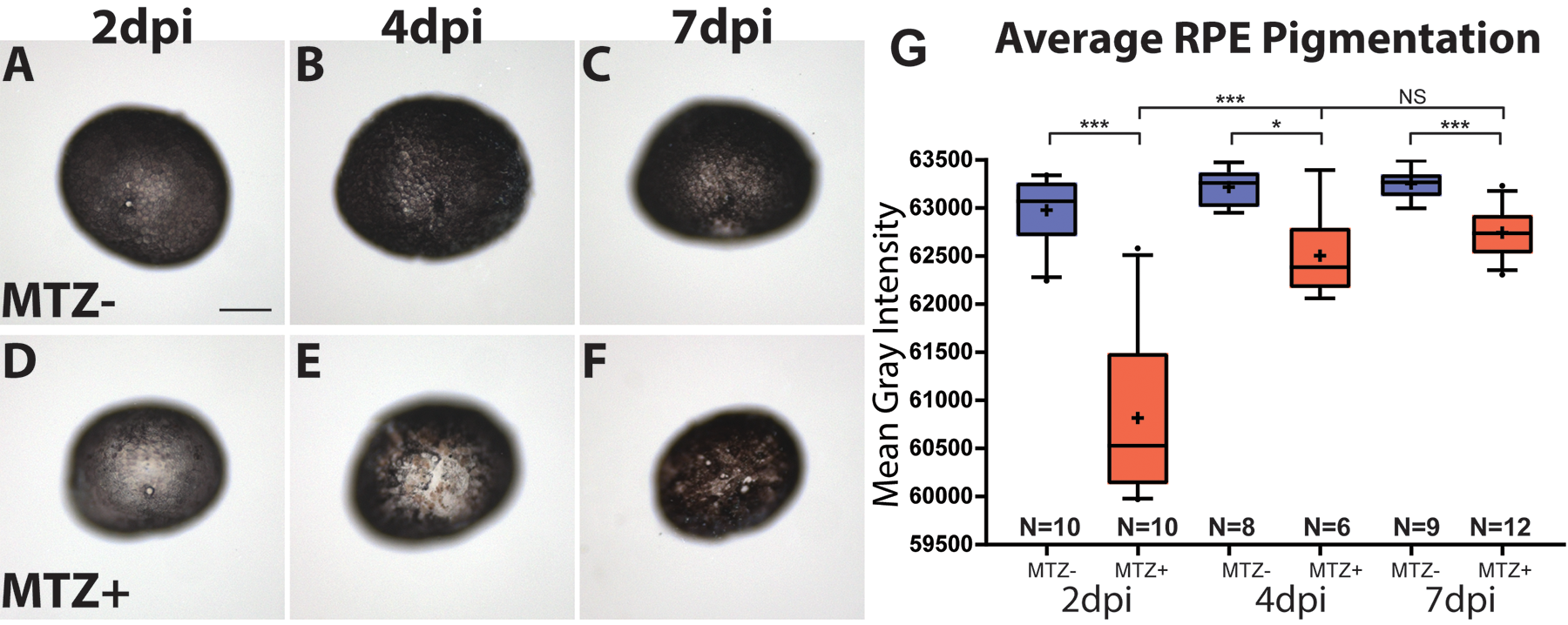

Supplement: S1 Fig — (A-F) Representative images of wholemounted eyes from unablated (A-C) and ablated larvae (D-F) at 2,4, and 7dpi. (G) Quantification of the mean gray intensity per eye reveals significant depigmentation in ablated larvae at 2dpi, and that pigmentation significantly improves between 2dpi and 4dpi, though overall pigmentation remains significantly reduced compared to unablated controls at 7dpi (Welsh’s t test, * P<0.05, **p<0.005, ***p<0.0005). (TIF) [file pgen.1007939.s001.tif]

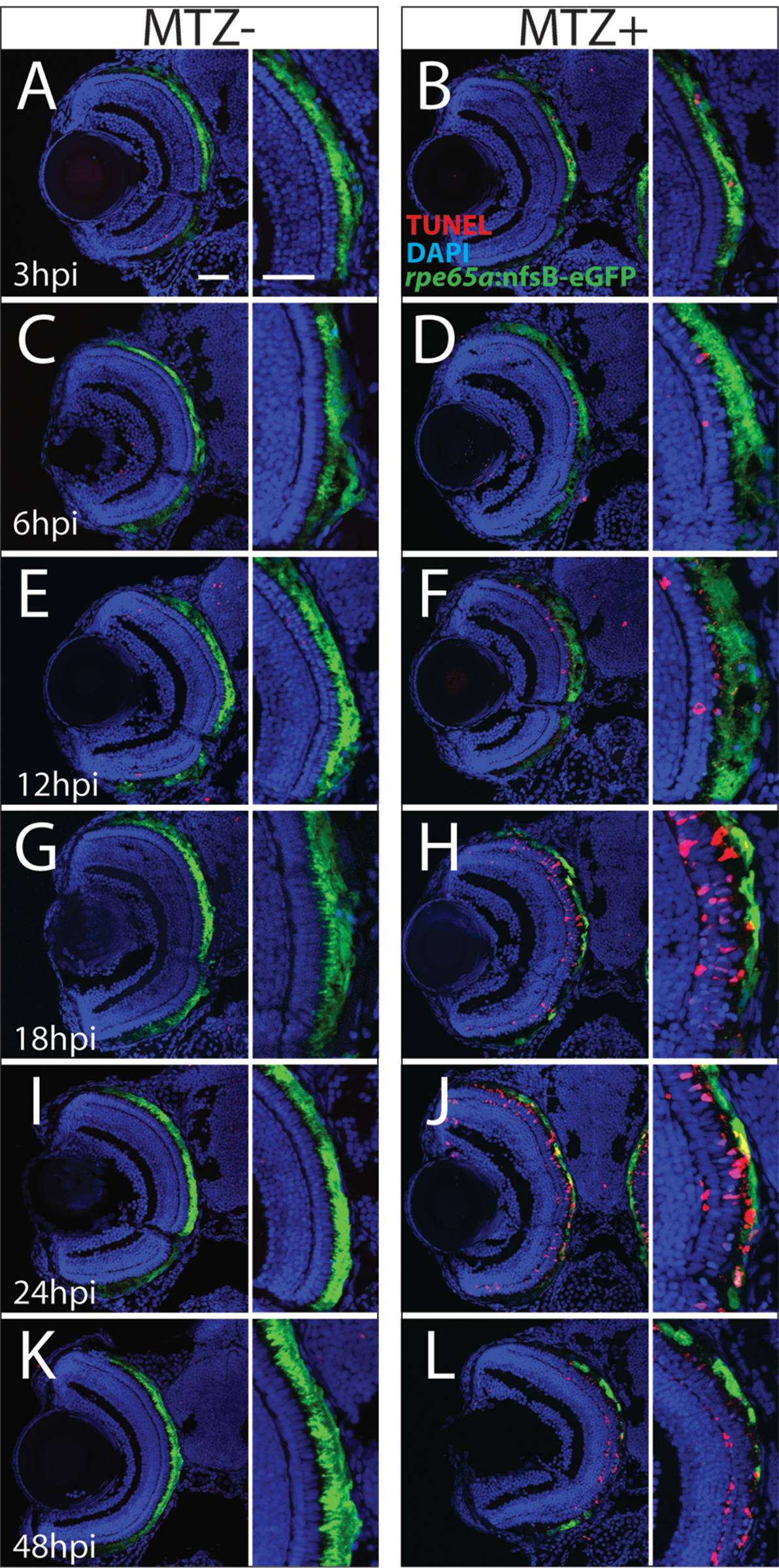

Supplement: S2 Fig — (A-L) Transverse cryosections stained for TUNEL (red). (A,C,E,G,I,K) Unablated and (B,D,F,H,J,L) ablated eyes at various time points following ablation. While the ONL appears to be unchanged at 3hpi, slight disruptions in ablated RPE morphology are detectible: apical microvilli become shortened compared to control, and the occasional TUNEL+ nucleus appears in the RPE layer (B). By 6hpi, degeneration of the eGFP+ apical microvilli and cell bodies becomes notable throughout the injury site, and nuclear organization in the ONL begins to degenerate (D). By 18hpi, eGFP signal begins to accumulate in blebs, leaving regions devoid of eGFP+ cells, and TUNEL signal appears throughout the RPE and ONL (H). Degeneration of the central injury site is complete by 48hpi, and TUNEL signal is reduced (L). (TIF) [file pgen.1007939.s002.tif]

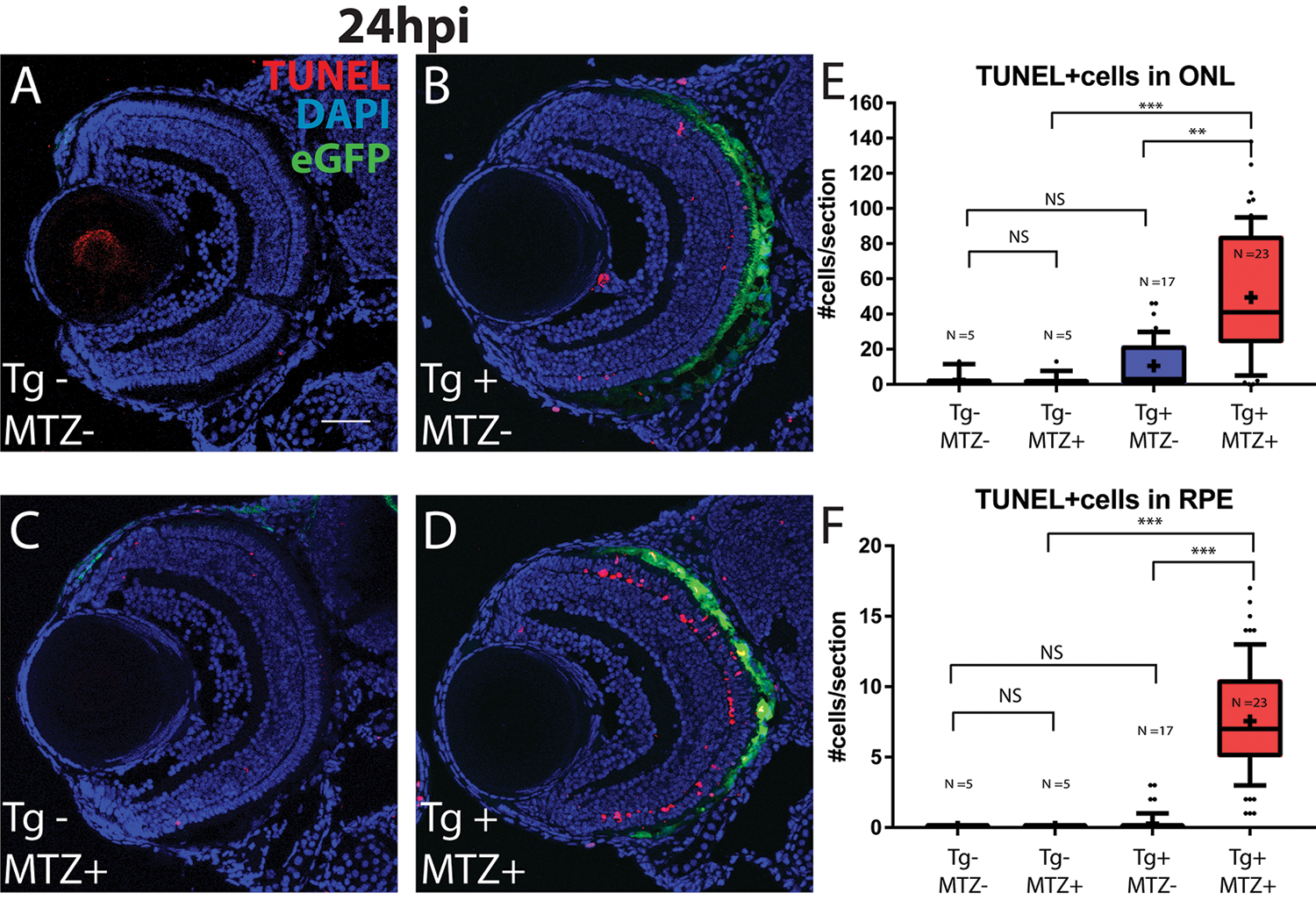

Supplement: S3 Fig — (A-D) Transverse cryosections stained for TUNEL (red). No TUNEL+ cells were detected in nontransgenic larvae (A,C) treated with and without MTZ. (E,F) Quantification of TUNEL+ cells/section in the ONL (E) and RPE (F). While ONL death appeared to be elevated in unablated rpe65a:nfsB-eGFP+ larvae, the increase was not significant (p = 0.1848), and no significant elevation of TUNEL+ cells was detected in the RPE (Mann-Whitney U test, *P<0.05, **p<0.005, ***p<0.0005). Scale bar = 40μm. (TIF) [file pgen.1007939.s003.tif]

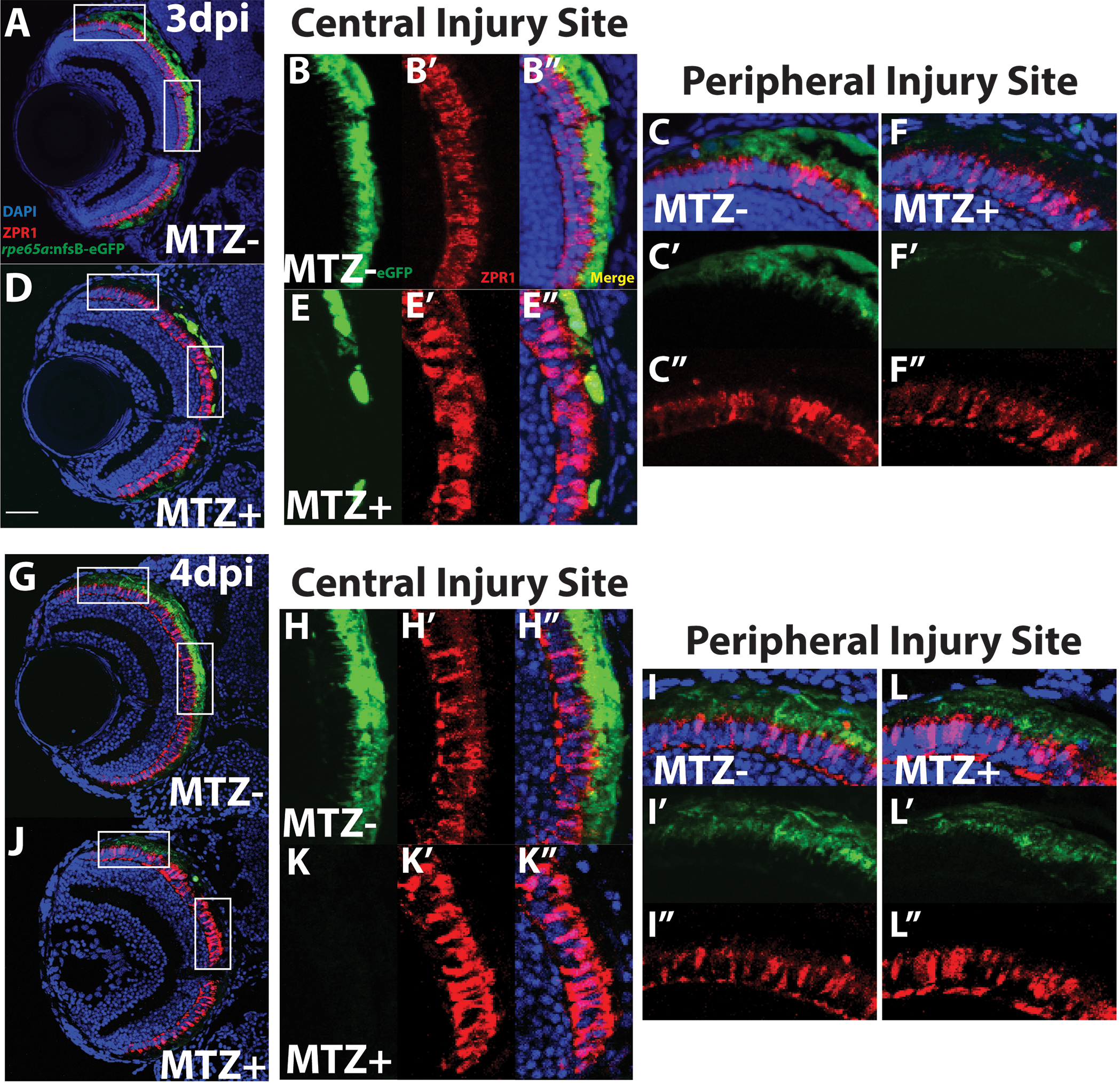

Supplement: S4 Fig — Transverse cryosections of unablated larvae (A,G) and ablated larvae (D,J) stained for ZPR2. Green = GFP, blue = nuclei, red = ZPR2. Magnified insets of central (B,E) and peripheral (E,F) RPE. (E-E”) At 3dpi, the ZPR1+ signal in PRs within the central injury site becomes less organized and reveals degeneration of outer segment tips. (F-F”) In the periphery, dim GFP signal marks the presence of regenerated RPE, and underlying ZPR1+ cones display morphologies similar to unablated controls. ZPR1+ cones closer to the periphery express normal levels of ZPR1 and identifiable outer segment tips, while those closer to the injury site display increasingly disorganized morphology and lack contiguous outer segment tips. (K-K” At 4dpi, ZPR1 signal continues to be degraded in the central injury site. (L-L”) In the periphery, where GFP+ RPE tissue has regenerated, ZPR1 staining is similar to unablated controls (I-I”), identifying organized cell bodies, outer segments, and outer segment tips. Scale bar = 40μm. (TIF) [file pgen.1007939.s004.tif]
